# Knockout of *Anopheles stephensi* immune gene *LRIM1* by CRISPR-Cas9 reveals its unexpected role in reproduction and vector competence

**DOI:** 10.1371/journal.ppat.1009770

**Published:** 2021-11-16

**Authors:** Ehud Inbar, Abraham G. Eappen, Robert T. Alford, William Reid, Robert A. Harrell, Maryam Hosseini, Sumana Chakravarty, Tao Li, B. Kim Lee Sim, Peter F. Billingsley, Stephen L. Hoffman

**Affiliations:** 1 Sanaria Inc. Rockville, Maryland, United States of America; 2 Insect Transformation Facility, Institute for Bioscience and Biotechnology Research, University of Maryland, Rockville, Maryland, United States of America; Pennsylvania State University, UNITED STATES

## Abstract

PfSPZ Vaccine against malaria is composed of *Plasmodium falciparum* (Pf) sporozoites (SPZ) manufactured using aseptically reared *Anopheles stephensi* mosquitoes. Immune response genes of *Anopheles* mosquitoes such as Leucin-Rich protein (LRIM1), inhibit *Plasmodium* SPZ development (sporogony) in mosquitoes by supporting melanization and phagocytosis of ookinetes. With the aim of increasing PfSPZ infection intensities, we generated an *A*. *stephensi LRIM1* knockout line, *Δaslrim1*, by embryonic genome editing using CRISPR-Cas9. *Δaslrim1* mosquitoes had a significantly increased midgut bacterial load and an altered microbiome composition, including elimination of commensal acetic acid bacteria. The alterations in the microbiome caused increased mosquito mortality and unexpectedly, significantly reduced sporogony. The survival rate of *Δaslrim1* mosquitoes and their ability to support PfSPZ development, were partially restored by antibiotic treatment of the mosquitoes, and fully restored to baseline when *Δaslrim1* mosquitoes were produced aseptically. Deletion of *LRIM1* also affected reproductive capacity: oviposition, fecundity and male fertility were significantly compromised. Attenuation in fecundity was not associated with the altered microbiome. This work demonstrates that LRIM1’s regulation of the microbiome has a major impact on vector competence and longevity of *A*. *stephensi*. Additionally, *LRIM1* deletion identified an unexpected role for this gene in fecundity and reduction of sperm transfer by males.

## Author summary

The Leucin-Rich Protein LRIM1 is an important component of the complement-like cascade in *Anopheles* mosquitoes and was shown previously to have anti-parasitic activity, supporting melanization and phagocytosis of *Plasmodium* ookinetes. To improve the manufacturing of Sanaria’s PfSPZ vaccine, by increasing Pf infection intensity in the mosquitoes, we have generated an *LRIM1* knockout line (*Δaslrim1*) using CRISPR-Cas9. Both bacterial load and the bacterial community composition altered significantly in *Δaslrim1*; these alterations were associated with reduced mosquito survival and, contrary to our expectations, dramatic reduction in Pf infection intensity. Reproductive capacity was reduced significantly in both females (fecundity) and males (fertility) of the *Δaslrim1* line. *Δaslrim1* mosquitoes cannot be used in the manufacturing of PfSPZ vaccine, but the data reveal novel and unexpected roles of *LRIM1* in different aspects of the mosquitoes’ vectorial capacity, such as survival and reproduction. Importantly, our findings demonstrate the importance of LRIM1 in controlling the microflora in the mosquitoes and thereby allowing successful infection of the parasites.

## Introduction

Sanaria PfSPZ vaccines, composed of aseptic, purified, cryopreserved Plasmodium falciparum (Pf) sporozoites (SPZ) are produced in aseptically reared *Anopheles stephensi* mosquitoes [1–5]. The cost of each dose of vaccine would be reduced significantly if more PfSPZ could be produced per mosquito. When Pf sexual stage parasites are ingested by *Anopheles* spp. mosquitoes, the parasites mate then transform to ookinetes which penetrate the mosquito midgut and form oocysts, which burst releasing PfSPZ into the hemocoel. After migrating to and invading the mosquito salivary glands, PfSPZ are inoculated into humans when the mosquitoes feed (reviewed by [6,7]). One way to increase PfSPZ production in mosquitoes would be to reduce innate immune responses in the mosquitoes [8–11] that are thought to inhibit parasite development.

*Anopheles* spp. mosquitoes possess innate immune systems that regulate microbial infections, including *Plasmodium* [10,12–15]. Invasion of the midgut epithelium by *Plasmodium* ookinetes activates a complement-like cascade initiated by a C3-homolog, thioester-containing glycoprotein 1 (TEP1), that circulates in the mosquito hemolymph [8]. Upon ookinete invasion of the midgut epithelium, TEP1 is cleaved by proteolysis. Cleaved TEP1 forms a complex with leucine-rich repeat (LRR) proteins, LRIM1 and APCL1, which is crucial for the stability of the cleaved form of TEP1 while circulating in the hemolymph [9,11]. The complex binds to the ookinete surface and labels it for melanization and phagocytosis [8,10,16,17]. Knock-down of *TEP1* by RNA interference (RNAi) resulted in a 5-fold increase in *P*. *berghei* (Pb) oocysts in the midguts of susceptible *A*. *gambiae* and elimination of melanization in a *P*. *berghei* refractory *A*. *gambiae* line, L3-5 [8]. Likewise, knock down of *LRIM1* or *APCL1* by RNAi led to an ~50-fold increase in oocysts in the midguts of susceptible *A*. *gambiae* and elimination of melanized parasites in a refractory *A*. *gambiae* [11].

Based on these findings, we hypothesized that deletion of *A*. *stephensi* immune genes would increase PfSPZ infection intensities. We have previously knocked down *LRIM1* by RNAi using the UAS-GAL4 system in which LRIM1 dsRNA was endogenously expressed [18].The knock-down resulted in 4-13-fold increase in midgut oocysts and 2-10-fold increase in salivary gland PfSPZ compared to WT [18]. Here, we used CRISPR-Cas9 to generate a stable *LRIM1* knockout line (*Δaslrim1*) of *A*. *stephensi*. Using this line, we have assessed the role of *LRIM1* in several important aspects of the mosquitoes vectorial capacity such as their longevity, fecundity, reproduction capacity and ability to support infection of *P*. *falciparum*. The *LRIM1* knockout line was dramatically more susceptible than the wild type (WT) to bacterial infection, but not more susceptible to PfSPZ infection. Our embryonic genome editing had an unexpected phenotype, profoundly reducing the reproductive capacity of the mosquitoes.

## Results

### Generation of LRIM1 knockout line in Anopheles stephensi, using CRISPR-Cas9

Multiple short guide (sg) RNAs (S1 Table) were used to target the first and the second exons on the 5’ end of *A*. *stephensi* LRIM1 (*aslrim1*) gene (Vector base—ASTE000814). The sgRNAs were mixed with recombinant CRISPR-associated protein 9 (Cas9) and injected into 549 *A*. *stephensi* embryos (S2 Table). Nineteen out of the 549 eggs hatched (3.5%), from which, only 4 females and 6 males developed to G_0_ adults. The adults were backcrossed *en masse* to WT males and females as appropriate. Females were provided with a blood meal and allowed to lay eggs. Twenty G_1_ egg pools were collected of which 8 groups of larvae were tested by PCR, using primers flanking the expected editing position (See primers 1 and 2 in S1A Fig and primers S6 Table). Sequence analysis of the PCR products revealed a 13 bp deletion in the 5’ of exon 2 (Nucleotides 942–954), in one group of G_1_ larvae (originating from one of the G_0_ males) out of the eight groups tested (S2 Table). The adults from the positive group in which the deletion was detected, were sorted and outcrossed to WT and after the blood feeding, the females were set to lay eggs individually. The other groups from which no editing was detected were discarded. Prior to egg laying, a leg was removed from each female, DNA was extracted from each leg and PCR was used to screen for females carrying the deletion. The G_2_ adults derived from the positive females were then in-crossed and individual females were allowed to lay eggs. Females were collected after egg laying and tested by PCR-sequencing, and G_3_ eggs were collected from positive females. Groups of G_3_ larvae_,_ derived from each of the positive G_2_ females, were tested by PCR with primers designed to detect alleles that were homozygous or heterozygous for the deletion (Primers 3 and 4 in S1A Fig). Groups of larvae with the highest frequencies of the deletion allele were continued on and the cycle repeated until a homozygous female was found in G_8_. The G_9_ larvae from that female were all homozygous, demonstrating that the null deletion was fixed in the mosquito line. PCR and sequence analysis on individual mosquitoes from G_12_ indicated that the deletion allele remained stable in the population in later generations (S1B and S1C Fig). Importantly, no evidence for the WT *LRIM1* allele was seen in any of the mosquitoes tested, indicating the establishment of a stable, homozygous *LRIM1* knockout line. This line will be referred to as *Δaslrim1* hereafter.

To confirm that the deletion resulted in silenced gene transcription, we performed qPCR on cDNA made from mRNA from different life stages of WT and *Δaslrim1* mosquitoes using two different reactions. In the first reaction the primers were targeting a region on the transcript that did not include the 13 bp deletion; therefore, this reaction should have amplified the fragment from both the WT and the *Δaslrim1* lines (S2A Fig, primers 5 and 6). In the second reaction, the 3’ end of the reverse primer was anchored in the deletion, which should not have resulted in amplification in the *Δaslrim1* line (Primers 7 and 8). Approximately 30% decrease in the total abundance of *LRIM1* transcript was observed in all tested life stages of *Δaslrim1* when qPCR was done with the first reaction (Primers 5 and 6), suggesting that while the transcript was still present, its abundance was somehow affected by the deletion. As expected, there was no amplification of *Δaslrim1* using the second qPCR reaction (Primers 7 and 8) in any of the different life stages, indicating that *LRIM1* is not expressed in the *Δaslrim1* line. The functional relationship between *LRIM1* and *TEP1* [17] prompted us to check whether transcription of *TEP1* was affected by the 13 bp deletion in *LRIM1* and/or its potential loss of function. No significant change was observed in TEP1 transcription TEP1 in all life stages of *Δaslrim1* compared to WT (S2A Fig).

The deletion of 13bp from the gene was predicted to cause a frame shift in the amino acid sequence of the protein at positions 57 to 62, which would potentially result in a stop codon, leaving only a small portion (62 amino acids) of the N-terminus of the protein. A polyclonal antiserum was raised against a 20 amino acid peptide in the middle of the LRIM1 protein, starting in position 84 which should not have reacted with any protein in *Δaslrim1*. Western blot analysis of hemolymph extracted from both WT and *Δaslrim1* demonstrated clearly that LRIM1 protein was not present in the hemolymph of the *Δaslrim1* line (S2B Fig).

### LRIM1 controls the midgut microflora and thereby longevity of *A. stephensi*

**Fig 1:**
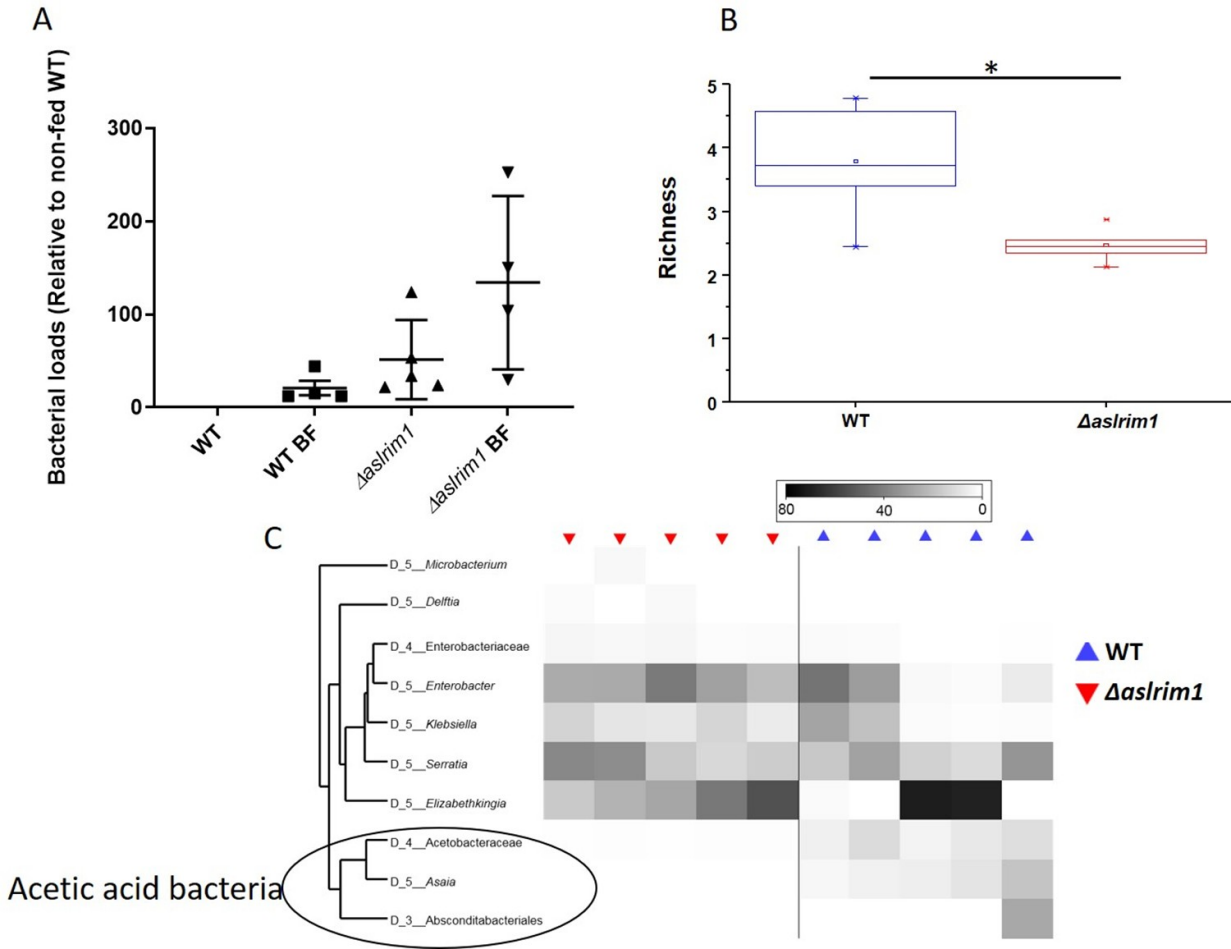
Effect of *LRIM1* deletion on the mosquito microflora. (A) Real-time PCR quantification of the bacterial population densities in the mosquitoes. The reaction used 16S rDNA primers, 515F and 806R (Primers 13 and 14, S6 Table), targeting the bacterial V4 region of the SSU rDNA [64]. The PCR was done on DNA from pools of 10 females from each mosquito line (WT and *Δaslrim1*) before and after blood feeding. The results represent the mean±SD of 5 replicates for *Δaslrim1* and 4 for the other samples. Mosquito ribosomal protein gene *S7* was used as housekeeping gene. (B) Alpha diversity richness analysis (to genus) in WT and *Δaslrim1* indicating higher microbial richness in WT relative *Δaslrim1* (Mann-Whitney U, P = 0.047). (C) Heatmap of the 10 most abundant microbial taxa across mosquito lines. Depth of sequencing ranged from 12827 to 65,296 sequences/sample (Mean = 38,682; median = 31,785).

We investigated the effect of *LRIM1* deletion on microbial populations in the mosquito midgut. First, we assessed the general bacterial loads in the mosquitoes using qPCR. Ten, non-fed female WT and *Δaslrim1* mosquitoes were collected and washed in 70% sterile ethanol and then twice in sterile PBS to prevent inclusion of bacteria from the mosquito surface in the extractions. DNA was extracted from individual mosquitoes and total bacterial load was analyzed by qPCR using 16S rDNA primers targeting variable region 4 (Primers 13 and 14, see S6 Table for primers) [19]. On average, bacterial load in *Δaslrim1* female was 21.3 ± 20.7-fold higher than in WT (S3A Fig). We repeated the experiment on WT and *Δaslrim1* before and after blood feeding. This time DNA was extracted from pools of 10 mosquitoes to minimize the high variability observed in the individual mosquitoes (Fig 1A). The total bacterial load in non-fed *Δaslrim1* was 52 ± 18-fold higher than in WT. Similar results were observed when using a culture-based approach when colony forming units (CFU) in *Δaslrim1* midguts were approximately 200-fold higher than the CFU in WT midguts (S5E Fig). The increase in the bacterial loads in *Δaslrim1* compared to the WT seemed to be more pronounced in the culture-based assessment compare to quantitative PCR. Differences between DNA- and culture-based approaches are not unexpected and are related to the ability of different bacteria to compete for the culture resources. Moreover, under different conditions, different bacteria are viable but nonculturable (VBNC) [20]. Finally, while in the DNA-based assessment we used DNA from pools of full mosquitoes, in the culture-based assay we plated only midguts from individual mosquitoes. In WT mosquitoes, blood feeding led to an increase in total bacterial loads by 21 ± 8-fold. Uptake of blood increased total bacterial load in *Δaslrim1* by 2.6-fold. However, the variability in the bacterial quantity in this line was high and the results between non blood fed and blood fed were not statistically significant (Fig 1A).

To evaluate the effect of the *LRIM1* deletion on the microbial community composition in the mosquitoes, the DNA pools from both lines were assessed by 16S ribosomal RNA (rRNA) gene amplicon sequencing [21], and bacteria identified to genus level (See dataset S1 for OTU). Raw reads (FASTQ files) were deposited at the NCBI Sequence Read Archive database (#PRJNA767345). Alpha diversity indices (calculated at a sequencing depth of 12,000 sequences/sample) indicated significantly lower microbial richness in *Δaslrim1* relative to WT (Fig 1B), but this phenomenon did not manifest itself in significantly different Shannon index values due to higher evenness in *Δaslrim1* samples (S4A and S4B Fig). Microbial community structure was significantly different between groups, as measured using analysis of similarity (ANOSIM) on Bray-Curtis similarity values (S4C and S4D Fig). The microbial communities in *Δaslrim1* mosquitoes were significantly different as compared to WT, and within-group *Δaslrim1* microbial communities were also more similar to each other than within-group WT communities (S4D Fig). Using DEApp software (https://www.ncbi.nlm.nih.gov/pmc/articles/PMC5291987/) DESeq2 analysis showed that 16 taxa were significantly different in abundance between groups, with 15 of them lower in *Δaslrim1* relative to WT (Fig 1C and Table 1). The bacteria most reduced in *Δaslrim1* mosquitoes were from the genera *Asaia* and *Tanticharoenia*, as well as an unidentified genus of *Acetobacteraceae* (Fig 1C). *Acetobacteraceae* are Gram negative bacteria that oxidize sugars to acetic acid during fermentation, some of which are known to be insect commensals, specifically of *Anopheles* mosquitoes [22–24]. A heatmap was generated of the 10 most abundant genera in WT mosquitoes and relative abundance compared with *Δaslrim1*. Proteobacterial genera (*Enterobacter*, *Klebsiella*, *Serratia*) and a genus of *Flavobacteriales* (*Elizabethkingia*) were variably abundant in all mosquitoes, but numbers were not significantly different between groups (Fig 1C). Again, the absence of the acetic acid bacteria could be clearly observed. Overall, deletion of *LRIM1* led to significant alterations in the mosquito midgut microbiome.

**Table 1:** Relative abundance of bacterial genera, *Δaslrim1* vs wild-type.

Taxon (Genus-Level)	Base Mean	Log2 Fold Change	Padj value
*Asaia*	2581.84	-14.56	6.38E-21
*Tanticharoenia*	166.62	-12.24	1.15E-09
*Caulobacter*	6.64	-7.58	5.90E-03
*Aquabacterium*	3.89	-6.82	1.57E-02
*Niabella*	2.73	-6.24	3.58E-02
*Enterococcus*	1.96	-6.09	3.87E-02
*Cellvibrio*	10.35	-6.08	4.24E-04
*Acetobacteraceae*	2100.01	-4.91	1.90E-09
*Variovorax*	21.36	-4.51	5.93E-04
*Deinococcus*	6.64	-4.28	8.09E-03
*Leptothrix*	7.61	-3.99	7.73E-03
*Sphingobacteriales*; env.OPS 17	15.76	-3.27	2.54E-02
*Sphingomonas*	12.97	-3.02	5.90E-03
Chloroplast	8.09	-2.99	3.32E-02
Unassigned	82.2	-1.99	3.40E-02
*Acetobacter*	2.62	4.17	4.25E-02

**Fig 2:**
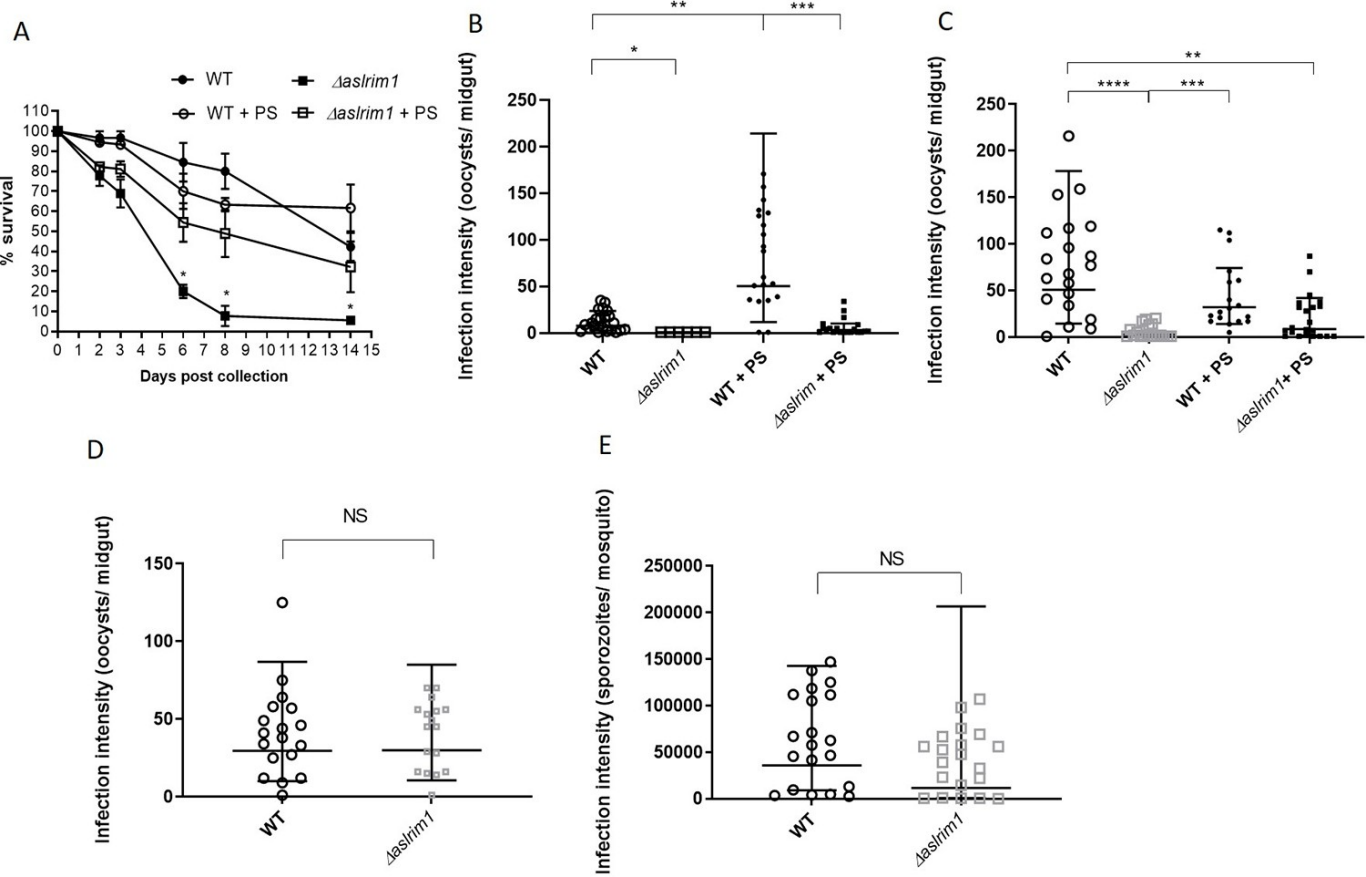
Effect of deletion of *LRIM1* on mosquito longevity and *Plasmodium falciparum* infection intensity. (A) Survival of WT and *Δaslrim1 A*. *stephensi* over a period of 14 days. Each point represents the mean ± SD of survival percentage in three different cages. The asterisks (p≤0.05) refer to the difference between WT and *Δaslrim1* grown with no PS, on days 6, 8 and 14. The data was analyzed with a non-parametric, Kruskal-Wallis and Dunn’s multiple comparisons tests. (B-C) Oocyst numbers in non-aseptic mosquitoes. Oocysts were counted by microscopy 7 days post bloodmeal for WT and *Δaslrim1* mosquitoes grown in non-aseptic conditions and fed on 15% sucrose with or without 1% penicillin/streptomycin (PS). The PS was added to the sugar meal for a short period (3- days prior to the bloodmeal) (B) or, throughout the entire adult life of the mosquitoes (C). Each point represents the oocyst number in a single midgut. (B) n = 22, 6, 20 and 20 for WT, *Δaslrim1*, WT + PS *and Δaslrim1* + PS, respectively. (C) n = 20, 19, 18 and 22 for WT, *Δaslrim1*, WT + PS *and Δaslrim1* + PS, respectively. The results were analyzed in non-parametric, Kruskal-Wallis and Dunn’s multiple comparisons tests. (D) The number of oocysts/midguts in WT and *Δaslrim1* mosquitoes grown under aseptic conditions, n = 18 and 17 for WT and *Δaslrim1*, respectively. The results were analyzed by the non-parametric, Mann Whitney test. (B-D) The results are expressed as a geometric means ±SD. Since some values were zero, a value of 1 was added to the entire dataset to allow calculation of the geometric means. (E) Number of sporozoites/mosquito in mosquitoes grown in aseptic conditions. The numbers of sporozoites were determined by dissecting the salivary glands of the mosquitoes and counting sporozoites from each mosquito by microscopy. The results are expressed as the geometric mean ± SD (n = 20 for both groups). The results for panel E were analyzed by the non-parametric, Mann Whitney test. For all panels, NS p>0.05, * p≤0.05, ** p≤0.01 *** p≤0.001, ****p≤0.0001.

We noticed high mortality of the *Δaslrim1* adults and hypothesized that this resulted from the high numbers of bacteria. We therefore monitored the mortality of WT and *Δaslrim1* mosquitoes with and without addition of penicillin-streptomycin (PS) in their sucrose meals. Providing 1% but not 0.5% PS eliminated internal bacterial populations from the WT mosquitoes [25,26] and did not compromise their survival and fecundity (S5A–S5C Fig). WT and Δ*aslrim1* were grown with PS treatment, the longevity of *Δaslrim1* mosquitoes increased while there was no effect on WT (Fig 2A). Thirty 3–4 day old mosquitoes untreated or treated with PS since emergence from pupae were collected and transferred to new cages (Day 0) and maintained on 15% sugar with or without 1% PS. The mosquitoes were blood fed three days after collection (Day 3). At that time point, over 90% of the WT mosquitoes were still alive whether they fed on regular sucrose or sucrose with PS. The survival of the *Δaslrim1* was only 69 ± 7% on the day of blood feeding. The survival of the *Δaslrim1* was improved (81 ± 4%) when PS was added to the sucrose meal. A big difference in the mortality was observed 6 days after the collection of the mosquitoes (3 days post bloodmeal), when only 20 ± 3% of the *Δaslrim1* were still alive compared to 84 ± 10% of the WT (P<0.05). The survival in *Δaslrim1* was partially rescued by feeding on PS, as 54 ± 10% of the mosquitoes were still alive at this point. A dramatic drop in the survival of *Δaslrim1* was observed 8- and 14-days post collection, when only 8 ± 5% and 6 ± 2% of the mosquitoes had survived respectively, compared to 80 ± 9% and 42 ± 7% survival of WT at these time points, respectively (P<0.05). Again, a significant, rescue of the survival in *Δaslrim1* was observed 8-and 14- days after collection, with the addition of PS to the sucrose meal. The results suggest a profound role for *LRIM1* in determining the longevity of the mosquitoes via controlling their internal microflora. The increased mortality of the mutants and its rescue by PS was observed in a second independent experiment (S3B Fig).

### LRIM1 is indirectly important for development of the Pf in non-aseptic *A. stephensi*

The complement-like cascade, and specifically *LRIM1*, are considered pivotal in the interactions between *Plasmodium* parasites and mosquito vectors. To assess the effect of the *LRIM1* knockout on growth and development of Pf, we infected WT and *Δaslrim1* mosquitoes, grown under normal (non-aseptic) and aseptic conditions, with Pf (Fig 2). Two to three day old WT and *Δaslrim1* adult mosquitoes were maintained non-aseptically on 15% sucrose ± 1% PS for three days, prior to blood feeding. Seven days post blood feeding, midguts were dissected and oocyst infection intensities were assessed (Fig 2B). In WT mosquitoes, the oocyst intensity was 7.9 geometric mean (GM) oocysts/midgut (95% confidence interval (CI) = 7.8–17.3), with a prevalence of 90%. Only 6 *Δaslrim1* female adults survived to 7 days post feeding and surprisingly, none were infected. Addition of PS to the sugar meal of the WT mosquitoes significantly increased the infection to 50.5 oocysts/midgut (95% CI = 57–105.3) and prevalence remained unchanged at 90% (Fig 2B). PS partially rescued the infection in *Δaslrim1* with an intensity of 3.2 (95% CI = 2.3–10.6) oocysts/midgut and prevalence of 60%. Due to the high mortality and the modest rescue by PS in *Δaslrim1* mosquitoes, we repeated the experiment starting with more mosquitoes and treating them with PS for their entire adult life, including throughout Pf development (Fig 2C). Oocyst infections were significantly lower in *Δaslrim1* (GM = 2 oocysts/midgut, 95% CI = 2.5–9.2) versus WT (GM = 50.8 oocysts/midgut, 95% CI = 52.3–104.8) (p<0.0001). Unlike the previous observation (Fig 2B), feeding on PS by WT mosquitoes reduced infection intensity slightly (GM = 32.2 oocysts/midgut, 95% CI = 26.4–61.4) and prevalence remained high (100%). Addition of PS to the sugar meal of *Δaslrim1* mosquitoes again partially recovered the infections (GM = 8.6 oocysts/midgut, 95% CI = 10.5–31.7, prevalence = 73%). In contrast, under aseptic conditions, the infection intensities between WT (GM = 29.6 oocysts/midgut, 95% CI = 27.3–56) and *Δaslrim1* (GM = 29.9 oocysts/midgut, 95% CI = 28.9–51.4) were almost identical and the prevalence was 94% for both lines (Fig 2D). A small, non-significant difference was observed in intensities of PfSPZ infections; 35,824 PfSPZ/mosquito (95% CI = 18768–68379) in the WT versus 11,590 PfSPZ/mosquito (95% CI = 23010–44630) in *Δaslrim1* (Fig 2E). The infection prevalence was 100% and 95% in WT and *Δaslrim1*, respectively. Overall, under normal growth conditions, oocyst infection intensities in *Δaslrim1* were significantly lower than in WT mosquitoes. This decrease was partially rescued by addition of PS to the sugar meal, and fully rescued when the mosquitoes were grown aseptically.

### A role for LRIM1 in mosquito fecundity and reproduction

**Fig 3:**
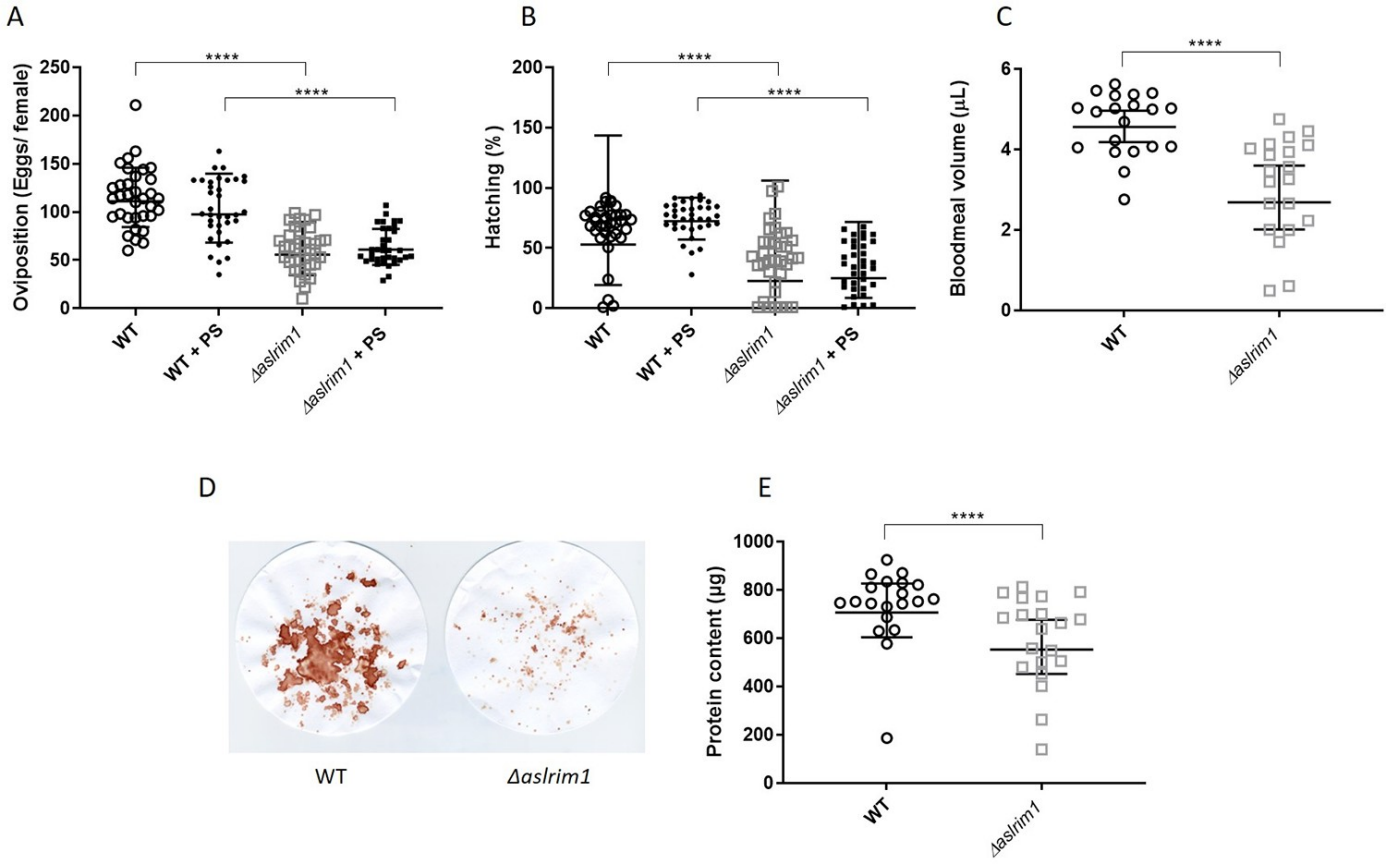
Fecundity in WT and *Δaslrim1* mosquitoes. (A) Number of eggs/ female. The number of eggs was determined by counting the eggs laid in single *Drosophila* tubes. The results are expressed as the geometric mean of the number of eggs/female ± SD; n = 34 for WT and 35 for the other 3 treatments. (B) Percentage of eggs hatching. The hatching was determined by counting the number hatching larvae in the single *Drosophila* tubes by microscopy, 2 days after egg laying. The percentage is of the total number of eggs laid in that tube (Panel A). The results are reported as the geometric mean of the percent hatching ± SD; n = 34 for WT and 35 for the other 3 treatments. Since some values were zero, a value of 1 was added to the entire dataset to allow calculation of the geometric means. (A and B) The data were analyzed by Kruskal-Wallis and Dunn’s multiple comparisons tests. **** p≤0.0001. (C) The bloodmeal volumes in WT and *Δaslrim1* mosquitoes immediately after blood feeding. The results represent the geometric mean of bloodmeal volumes ± SD; n = 20. (D) Remnants of blood on the filter paper after blood feeding of 50 females, as an indication of prediuresis. (E) Bloodmeal protein contents in midguts of WT and *Δaslrim1* mosquitoes, immediately after blood feeding. The protein contents were determined using Lowry protein assay. The results represent the geometric mean of midgut protein contents in micrograms ± SD; n = 20. (C and E) The results were analyzed using non-parametric, Mann Whitney test. ** P≤0.01, ****P≤0.0001.

As there was a profound attenuation in the reproductive capacity of *Δaslrim1* compared to their WT counterparts, we examined fecundity (egg production and egg hatching rate) in females and fertility in males. WT and *Δaslrim1* mosquitoes were provided with 15% sucrose ± 1% PS from the day of adult emergence. Females were provided with a bloodmeal 1 week post emergence and engorged females were separated into cages. Three days post bloodmeal, females from WT, WT+PS, *Δaslrim1* and *Δaslrim1*+PS, were placed individually into *Drosophila* tubes and allowed to oviposit. The number of females that laid eggs was determined and the eggs were counted in 34–35 tubes collected randomly from each group; the results are summarized in Fig 3A and S3 Table. The majority of WT females had laid eggs, 92% and 95% of the WT and WT+PS females, respectively while only 61% and 55% of the *Δaslrim1* and *Δaslrim1*+PS, respectively, had oviposited. The number of eggs laid by individual *Δaslrim1* females was reduced significantly to 55.7 (95% CI = 47.2–65.8) and 60.9 (95% CI = 54.9–67.6) in the absence and presence of PS, respectively, from WT 111.1 (95% CI = 104.2–126.0) and 97.6 (95% CI = 92.3–114.1) in the absence or presence of PS, respectively (Fig 3A and S3 Table). The observation of the reduction in oviposition in the mutants which was not rescued by addition of antibiotics was shown in an additional experiment (S4 Table). Thirty females of both WT and *Δaslrim1* mosquitoes were put together in 6 cages for each line and reared to adults on 15% sugar, with or without 1% PS. A cup of water was placed in the cages and mosquitoes were allowed to oviposit. The number of eggs in each cup was determined and divided by the number of surviving adults on the day of oviposition. Egg laying in this experiment was low compared to our other observations, WT female laid on average 14.4 ± 11.9 eggs. WT mosquitoes grown with PS laid 29.5±15.4 eggs. Eggs were not found in either one of the *Δaslrim1* cages, with or without PS despite the fact that the low survival rate in the mutants was rescued by addition of PS (S3B Fig). Deletion of *LRIM1* also resulted in a significant decrease in hatching rates (Fig 3B and S3 Table). In WT mosquitoes, the mean egg hatching rate was 52.9% (95% CI = 37.5–74.5) and 72.4% (95% CI = 66.7–78.6) in WT and WT + PS, respectively. In *Δaslrim1*, egg hatching was significantly lower (p<0.0001) at 22.8% (95% CI = 13.5–38.7) and 25% (95% CI = 17.5–36) for *Δaslrim1* and *Δaslrim1* + PS, respectively. Altogether, deletion of *LRIM1* resulted in a significant reduction in fecundity in terms of oviposition rate, number of laid eggs per female and egg hatching rate. None of these were rescued by the addition of PS to the sucrose meal, suggesting that low fecundity in *Δaslrim1* was not associated with the increased bacterial loads. To determine whether the low number of eggs was due to a reduction in blood intake by the *Δaslrim1* mosquitoes, females were randomly collected immediately after the bloodmeal and the volume of bloodmeal was determined). The geometric mean volume of bloodmeal taken by WT mosquitoes was 4.6 μL (95% CI = 4.2–5.0 μL) while in *Δaslrim1* females it was only 2.7 μL (95% CI = 2.0–3.6 μL) of blood (1.7-fold less, P<0.0001) (Fig 3C). *Anopheles* mosquitoes concentrate host blood cells and proteins, simultaneously excreting excess salts and water, in a process called prediuresis which is exemplified by the release of large blood-colored droplets during feeding [27,28]. During rearing of the mosquitoes, we noticed a dramatic reduction in prediuresis products in Δ*aslrim1* mosquitoes compared to WT. To demonstrate this difference, we transferred 50 WT and Δ*aslrim1* females each to a 473 mL cardboard container and placed a round filter paper at the bottom of the container. After feeding, more prediuresis products were seen on filter papers from the WT containers compared to filter papers from the Δ*aslrim1* containers (Fig 3D), indicating that bloodmeal processing is severely disrupted by the *LRIM1* deletion. Total blood meal protein was measured in both lines to determine if the difference in prediuresis was also manifested in lower acquisition of proteins from the bloodmeal (Fig 3D). The geometric mean blood meal protein content/midgut in Δ*aslrim1* was 553 μg protein/midgut (95% CI = 451.8–676.1 μg protein/midgut) compared to 706 μg protein/midgut (95% CI = 604.0–826.5 μg protein/midgut) in the WT. To reinforce that the decrease in blood intake, which had potentially contributed to the reduction in fecundity, was due to LRIM1 deletion, we have outcrossed *Δaslrim1* female from G_9_ to WT males and reestablished a new homozygous *Δaslrim1* line using the same molecular tools that were used to establish the original line. Blood intake and protein contents were compared again between WT and the new *Δaslrim1* line (S3C and S3D Fig). The results were consistent with our previous observation. In this experiment, both lines ingested less blood compared to those in the first experiment, but the difference between the two lines remained the same (1.7-fold less blood in the mutants). Bloodmeal protein contents were also significantly lower in the mutants compare to WT, consistent with our initial observations.

### LRIM1 has a role in male fertility

**Fig 4:**
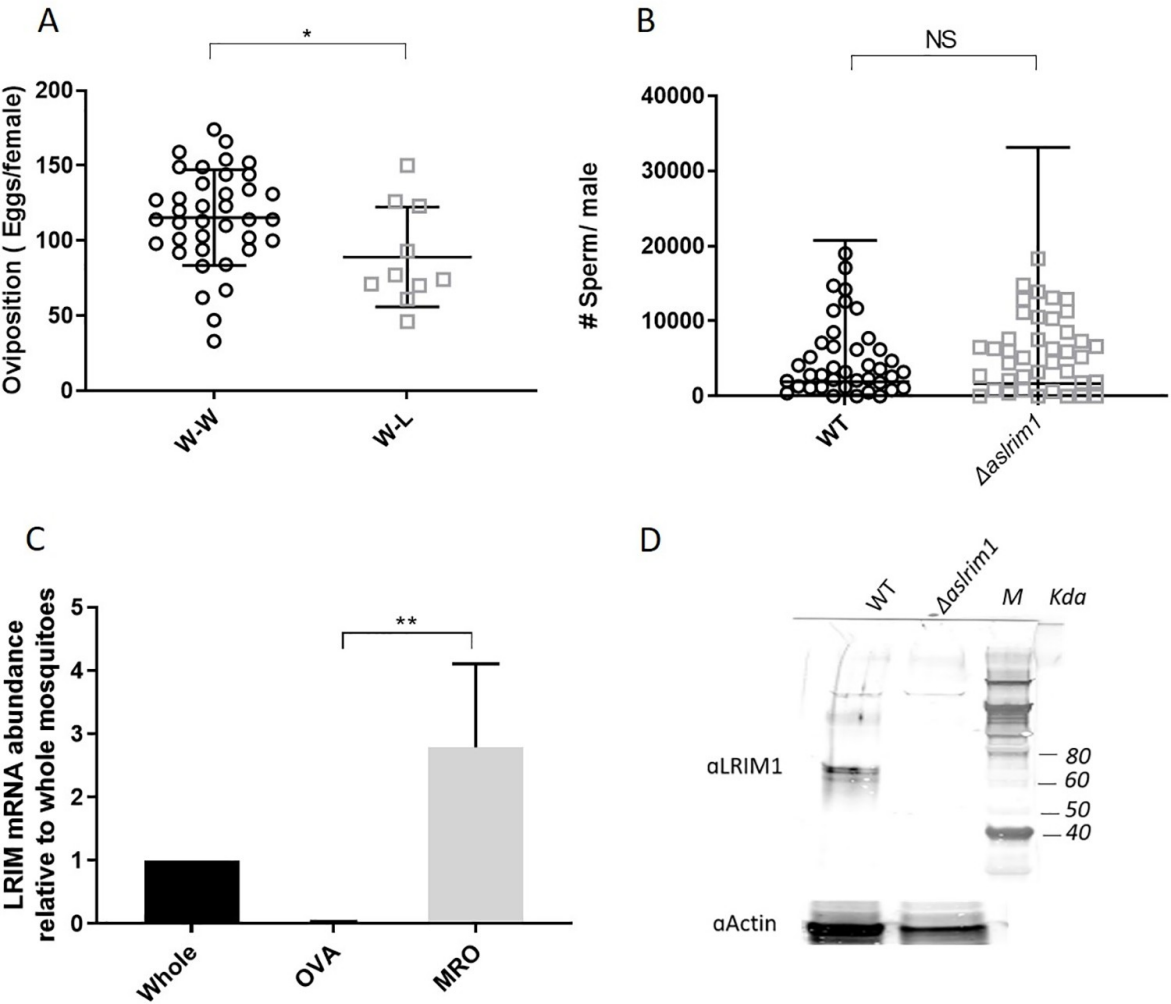
Effect of deletion of *LRIM1* on fertility in male mosquitoes. (A) Number of eggs laid in single *Drosophila* tubes by WT females, mated with WT (W-W) or *Δaslrim1* (W-L) males. The results are the mean number ± SD (n = 38 and 10 for W-W and W-L, respectively). Results were analyzed by unpaired t-test. (B) The number of sperm/male was determined by dissecting the male testes and accessory glands from each male and counting the number of sperm in a hemocytometer under the microscope. The results are presented as the geometric mean ± SD (n = 40 and 31 for WT and *Δaslrim1* respectively) and are representative of 2 independent biological repeats with similar results. The data were analyzed using non-parametric, Mann Whitney test. Since some values were zero, a value of 1 was added to the entire dataset to allow calculation of the geometric means. (C) Relative abundance of *LRIM1* mRNA in ovaries (OVA) and male reproductive organs (MRO) of WT mosquitoes was determined by real-time PCR using primers specific to *LRIM1* (Primers 5 and 6, S6 Table). The abundance was normalized to the mosquito *S7* ribosomal protein gene (Primers 9 and 10, S6 Table). ** P≤0.01. The results shown here are representative of two independent biological repeats, using RNA extracted on two different occasions. (D) Western blot analysis done on pools of male reproductive organs (MRO) from 30 WT and *Δaslrim1* male mosquitoes. The proteins were transferred to PVDF membrane and reacted with 1:250 rabbit polyclonal anti LRIM1, generated in this work. Beta actin (1:1000) was used as a loading control.

The significant decrease in oviposition and hatching rate in *Δaslrim1* mosquitoes could be due to reduced fertility of *Δaslrim1* males. To assess whether the deletion of *LRIM1* had an effect on male fertility, WT female pupae were sorted and crossed *en masse* with either WT (W-W) or *Δaslrim1* (W-L) males. Mosquitoes were reared to adults, females were provided with a bloodmeal, and the number of eggs per female and the proportion of fecund females were determined. The hatch rate was determined 3-days post egg laying. The females that did not lay eggs were dissected and examined microscopically for the presence of eggs in the ovaries and sperm in the spermatheca (Table 2). Bloodmeal size was the same in W-W and W-L females, confirming that blood intake was not a factor in oviposition outcome or the number of eggs. In the W-W, 60.3% of the females laid eggs compared to only 14.3% in W-L, consistent with the reduced percentage of egg laying females observed previously in *Δaslrim1* (S3 Table). The mean fecundity in W-L was moderately but significantly (P<0.05) lower than that of W-W cross (89.1 ± 10.5 eggs/female and 115.3 ± 5.2 eggs/female, respectively) (Fig 4A). An equal proportion of females that did not lay eggs in W-W and W-L, had eggs in the ovaries (47.1% and 46.7%, respectively). Sperm were detected from the spermathecae of 52.9% of females that did not lay eggs in W-W and only 22.2% in W-L. These data suggest that Δ*aslrim1* males have a reduced capacity to inseminate females or that the number of sperm that they deposit in the females is below the detection level. However, *Δaslrim1* and WT males had similar numbers of sperm in their testes (Fig 4B). These results were consistent with the results in a second experiment in which only 9% of the WT females that were crossed with *Δaslrim1* males (W-L) had laid their eggs compared to 32% of the females that were crossed with the WT males (W-W) (S5 Table). In the females that did lay eggs, the number of eggs/female did not change.

**Table 2:** Egg production in WT females and oviposition following a cross with *Δaslrim1* or WT males.

Cross	#Live males^2^	#Live females		% Females laying eggs	% Females with eggs in the ovaries*	% Females inseminated *
		Feeding^1^	Egg laying^2^			
*♂*WT X *♀*WT	63	43	27	60.3	47.1	52.9
*♂Δaslrim1* X *♀*WT	70	33	12	14.3	46.7	22.2

* Percentage is calculated from the number of females that did not lay eggs.

^1^ The numbers were determined at the day of blood feeding.

^2^ The numbers were determined at the day of egg laying.

LRIM1 protein is expressed in the hemolymph of *A*. *stephensi* and *A*. *gambiae* (S2B Fig)[11], and in the midgut of *A*. *gambiae*, 24–48 hours following an infected bloodmeal [29]. Due to the effect of *LRIM1* deletion on both fecundity and male fertility, we looked for LRIM1 expression in the reproductive organs of the mosquitoes. RNA was extracted from pools of male reproductive organs (MRO—testis, male accessory glands, *vas deferens* and ejaculatory duct) and ovaries, from 30 sugar-fed 4-6-day old male and female mosquitoes, respectively. The *LRIM1* mRNA abundance in those tissues relative to whole mosquitoes was assessed using real-time PCR with *LRIM1* specific primers (Primers 7 and 8 S6 Table). *LRIM1* mRNA was significantly more abundant in the male reproductive organs compared to whole mosquitoes. Conversely, we did not detect *LRIM1* mRNA in the ovaries (Fig 4C). Western blot analysis indicated expression of LRIM1 protein in reproductive organs from WT but not *Δlrim1* males.

## Discussion

We generated *LRIM1* knock out (*Δaslrim1*) *A*. *stephensi* to reduce the innate immune responses in the midgut against Pf ookinetes and oocysts, with the aim of improving PfSPZ numbers for manufacturing PfSPZ products. Using CRISPR-Cas9, we were able to generate a deletion in the *LRIM1* coding region that completely prevented expression of LRIM1 protein. Deletion of the *LRIM1* gene had a profound impact on the quantity and diversity of the mosquito midgut microbiome, increasing total bacterial load and reducing midgut microflora diversity and richness. The *Δaslrim1* mosquitoes were colonized predominantly by known mosquito commensals such as the proteobacteria *Enterobacter*, *Klebsiella*, *Serratia* and *Flavobacteriales*, as well as by *Elizabethkingia* but other classes, such as the acetic acid bacteria, were lost from the midgut. The loss of the acetic acid bacteria and specifically the *Asaia* genus, is of particular interest because it is an important commensal bacterium that is highly abundant in *Aedes* and *Anopheles* species, particularly *A*. *stephensi* [22,30–32]. The *A*. *stephensi* abdomen is colonized predominantly by different *Asaia* species which account for 41%, 25% and 20% of the total population in the gut, salivary glands and female reproductive system, respectively [30], and 58% of the bacterial population in the male reproductive system. The changes in the mosquito microbiome and the bacterial overgrowth in *Δaslrim1* mosquitoes significantly reduced their survival, indicating the pivotal but probably indirect role of *LRIM1* in mosquito longevity.

Contrary to our expectations, PfSPZ infection intensities did not increase in *Δaslrim1*. Under normal rearing conditions, there was a dramatic reduction in PfSPZ intensities which was partially rescued by treating the mosquitoes with antibiotics, and completely rescued when mosquitoes were reared under aseptic conditions. *LRIM1* appears crucial for the development of Pf in non-aseptic *A*. *stephensi*, most likely by regulation of the mosquito microflora. Therefore, establishing infection and completion of the sporogonic cycle by the parasite, depends heavily on the interactions between the parasite and the internal microflora. The concept of parasite-microbiota interactions inside the mosquito is not new and the antiparasitic effect of bacteria has been demonstrated for a variety of *Anopheles* and *Plasmodium* species [33]. Most of these studies suggest that microbiota-related parasite killing is achieved mainly by the stimulation of the mosquito immune response by the bacteria [34–37]. However, the results of our work, specifically the overwhelming increase in bacterial loads in *Δaslrim1* mosquitoes, strongly suggest that under natural, non-aseptic conditions competition for nutrients and/or reduced fitness due to increased bacterial load also plays a major part in determining the ability of the parasite to develop optimally in the mosquito. Whether the anti-parasitic effect of the microbiota is through stimulation of the immune response or simply by competition for resources or reduced mosquito fitness, *LRIM1* is important in controlling the microbiome and thereby providing the parasite with an environment in which it can flourish.

The decrease in PfSPZ infection intensity following the *LRIM1* deletion was contrary to what had been reported by us and by others, showing an increase in either *P*. *berghei* (Pb) and Pf oocyst or PfSPZ numbers upon the knock-down of *LRIM1* by RNAi [11,17,18]. The differences are likely due to the different genetic manipulation approaches that were taken. Unlike in gene knockouts, RNAi does not lead to a complete elimination of the targeted proteins. In line with this, reduced expression through RNAi of a fibrinogen-related protein 1 (*FREP1*) resulted in only partial protein depletion and subsequently, partial reduction in midgut oocyst infections (50%) [38]. In contrast, complete knockout of *FREP1* protein by CRISPR-Cas9 resulted in a much stronger suppression of the infection (~80%) [39]. Moreover, the knock-down of *LRIM1* in *A*. *stephensi* by RNAi resulted in a significant increase in Pf infection intensity [18]. However, in that work, at least 40% of the *LRIM1* transcript was still present at all life stages tested. Importantly, the knock-down did not affect the bacterial load in the mosquitoes and this is likely to be the main reason for the differences between the knock-down of *LRIM1* and its complete elimination by CRISPR-Cas9 in the present work. In other studies, *LRIM1* dsRNA was injected into 1-2-day old adult *A*. *gambiae* which were infected a few days later [11,17], probably too short a time frame for the excessive bacterial burdens to establish compared to the present work, where the *LRIM1* deletion was permanent, affecting mosquitoes right from the early stages of development. In summary, while we cannot rule out that *LRIM1* and the complement-like system interacts directly with the parasites as suggested previously, the comparable infections in aseptically reared WT and *Δaslrim1* mosquitoes suggest that the effect of *LRIM1* on Pf infection is indirect and mediated by the mosquito microbiota.

Deletion of *LRIM1* significantly reduced the fecundity and reproduction of the *Δaslrim1* mosquitoes which could not be rescued by addition of antibiotics to the mosquito sucrose meal. Notably, addition of PS to the mosquito sugar meals did not completely rescue the bacterial load in the mutants (S5E Fig, *Δaslrim1* + PS) and therefore we cannot rule out that the altered microbiome had a role in the compromised fecundity. However, PS did reduce the bacterial load in the mutants and therefore it is reasonable to expect some level of rescue, which we did not see. Therefore, we conclude that *LRIM1* has a role in fecundity that is independent of the mosquito microbiota. This reduction in fecundity maybe explained at least in part by the reduction in blood intake and in prediuresis by *Δaslrim1* females. A direct role of *LRIM1* in oogenesis is yet to be defined even though there is some evidence for an association between oogenesis and the complement-like system. Werling et al., [40] found a positive correlation between eggs and oocyst development. They suggest that the steroid hormone 20-hydroxy ecdysone that promotes accumulation of lipids and other nutrients in the ovaries which are essential for oogenesis, also controls TEP1-mediated parasite killing. In line with this, Vitellogenin (Vg) is a nutrient transporter, essential for delivering digested bloodmeal peptides to the maturing mosquito oocytes [41,42]. Disruption of Vg resulted in defects in egg development and in parallel, significant reduction in *TEP1*-mediated ookinete killing [43]. MosGILT, a mosquito saliva protein, is also involved in oogenesis and its knock-down led to a profound impairment in ovarian development that was coupled with significant reduction in TEP1-mediated parasite killing [44]. In another study, deletion of *FREP1*, a *Plasmodium* agonist, in *A*. *gambiae*, led to a significant reduction in infection intensities by both *P*. *berghei* and Pf but also had a profound fitness cost in which blood ingestion, fecundity and egg hatching all decreased significantly [39], supporting the premise of association between parasite infection and reproductive capacity.

In *Anopheles*, oviposition depends heavily on the male’s ability to inseminate females and fertilize the eggs [45,46]. Thus, the reduction in egg laying might also be explained by the effect of LRIM1 deletion on the fertility of the males and their ability to fertilize the eggs. Despite the fact that LRIM1 is expressed in the male reproductive organs, sperm counts in *Δaslrim1* males were comparable to those in WT. Therefore, the role of *LRIM1* in male fertility is not based on the ability of the males to produce sperm but rather on the quality of the sperm, the seminal fluids, and/or on the ability of the males to inseminate the females. This premise is supported by the observation that when WT females were crossed with *Δaslrim1* males the proportion of ovipositing females decreased profoundly but on the other hand, the number of eggs / female or percent hatching in the females that did lay eggs, did not change significantly. This suggests that reduced fertility in *Δaslrim1* males results in the reduction in proportion of ovipositing females, while reduction in egg production in the *Δaslrim1* females is related to a role for *LRIM1* in oogenesis. This hypothesis will have to be addressed by crossing WT males with Δ*aslrim1* females. The involvement of the complement-like system in determining the quality of the sperm was demonstrated previously [47]; in *A*. *gambiae*, TEP1 binds to the surface of damaged sperm in the testes, labeling them for removal and thereby allowing for a high rate of healthy sperm production. In this context, the supporting role of *LRIM1* in stabilizing the active form of TEP1 [9,17] may also be relevant in maintaining sperm quality.

It is interesting to note that acetic acid bacteria, and specifically *Asaia*, that were removed from the microflora in *Δaslrim1* mosquitoes, typically populate the reproductive organs of male and female *A*. *gambiae* and *A*. *stephensi* [22,24,30]. Moreover, in these mosquitoes, *Asaia* is transmitted horizontally from males to females through mating and vertically from the female to the eggs [30,48]. We do not have evidence for an association between the elimination of bacteria species such as *Asaia* and the low fecundity in *Δaslrim1*, but it is possible that the regulation of internal microbiota by *LRIM1* allows important commensal bacteria such as *Asaia* to successfully colonize the reproductive organs, and in the absence of LRIM1 protein, such regulation may be disrupted resulting in overgrowth of other species.

The unexpected reduction in mosquito fecundity following *LRIM1* deletion was independent of the role of *LRIM1* in controlling the midgut microbiota, inferring that *LRIM1* has an additional, unidentified role in the reproductive capacity of the mosquitoes. This demonstrates the power of precise CRISPR-Cas9 genome editing to reveal novel functions of targeted genes while raising serious concerns about our ability to accurately attribute specific biological functions to a gene. Such unexpected phenotypes are important considerations for embryonic gene editing by CRISPR-Cas9 in other species, including humans, where creation of adverse phenotypes might not be manifested until later in life [49–51].

Random, off-target genomic editing is one known limitation of the CRISPR-Cas9 technique. Moreover, bottleneck selection may have occurred in the mosquitoes that were recovered from the embryonic injections. We cannot rule out the possibility that these phenomena may have contributed to some if not all of the phenotypes observed in this work. However, during the establishment of the line, in each generation the mosquitoes were selected based on the 13 bp deletion and therefore, unless genetically linked, random off-target editing were most likely out selected. Moreover, the mosquitoes recovered from the embryonic injections (G_0_) as well as the following generation (G_1_) were backcrossed to WT. These consecutive backcrosses had most likely resulted in dilution of potential bottlenecks or CRISPR-related off-target effects. Finally, reestablishing new *Δaslrim1* line by outcrossing Δ*aslrim1* female from G_9_ to WT males, following multigenerational in crossings resulted again in reduced blood intake and bloodmeal protein content. This reinforce the premise that this phenotype is a result of *LRIM1* deletion.

Our goal was to increase PfSPZ yields in *A*. *stephensi* and thereby improve PfSPZ production by deleting the gene encoding the *A*. *stephensi* immune deficiency protein, LRIM1. The resultant mutant line, *Δaslrim1*, was significantly more susceptible to bacteria but not to Pf and moreover, was severely compromised in reproductive capacity. Therefore, this line of mosquitoes will not be useful for the manufacturing of PfSPZ vaccines. We are currently testing the possibility of improving PfSPZ yields by deleting other innate immune responses genes.

## Materials and methods

### Mosquito and parasite growth and infection

The mosquitoes used in this study were *Anopheles stephensi* SDA500, reared at the University of Maryland Insect Transformation Facility (ITF) at the Institute for Bioscience and Biotechnology Research (IBBR), using standard conditions (28°C and 75% humidity). For aseptic rearing of mosquitoes, egg laying was induced and eggs were transferred to Sanaria for aseptic production[1]. For infection of mosquitoes, Pf NF54 strain were grown in blood culture for 18–20 days for the development of gametocytes. Mosquitoes were then fed through a membrane with infected blood (5 million gametocytes/mL), as established previously [1,52,53].

### Mosquito rearing, CRISPR mix and Injection into A. stephensi embryos

CRISPR mix was done based on the protocol of Kistler et al. [25]. Briefly, short-guide (sg) RNAs were generated by PCR amplification of the guides following by in vitro transcription using the Ambion Megascript T7 kit (#AM1334) according to the manufacturer instructions. The RNAs were purified using Megaclear Transcription Clean-Up kit (Thermo Fisher #AM1908). Multiple sgRNAs (40 ng/μL) were mixed with 300ng of recombinant CAS9 (PNA bio—CP01). CRISPR injection mix was injected by standard methods [54,55] into preblastoderm *A*. *stephensi* embryos. Embryos were injected between 40 min (TS) and 60 min (TF) post start of embryo collection, with T0 being the time mosquitoes were added to egging chamber, Ts being average start time of injection, and TF being average time of completion for the last embryo of an injection round. All injections were done at the University of Maryland ITF at IBBR.

### DNA and RNA assessments

Adults or larvae of *A*. *stephensi* mosquitoes were homogenized using blue pestles and genomic (g)DNA was extracted from the homogenates using Qiagen DNeasy Blood & Tissue Kit (#69506). RNA was extracted from mosquito homogenates using Qiagen RNeasy mini kit (#74106) according to the manufacturer instructions. PCR reactions were done using MyTaq red DNA polymerase (bioline USA #BIO-21108). Complimentary (c)DNAs were synthesized using High-Capacity cDNA Reverse Transcription Kit (Thermo Fisher # 4368814) on extracted RNAs. Real-time PCR reactions were done on cDNAs or gDNAs (for assessment of bacterial loads) using SensiFAST Real-Time PCR Kits (Bioline # BIO-82005).

### Production of LRIM1 antibody and western blot analysis

Polyclonal αLRIM1 antiserum was produced by injecting a short peptide (KLH conjugated), corresponding to amino acids 437–456 of A. *stephensi* LRIM1 protein into rabbits (Done by Envigo Bioproducts). For western blots, mosquito hemolymph was extracted as described previously [11] and male reproductive organs (testes, male accessory glands, *vasa deferentia* and ejaculatory ducts) were each pooled from 30 mosquitoes and lysed in RIPA lysis and extraction buffer (Thermo Scientific #89900) according to the manufacturer’s instructions. Samples were run on SDS-PAGE gel and probed with the polyclonal αLRIM1 antiserum at 1:250 dilution, then a 1:5000 secondary anti rabbit (AP conjugated).

### Survival, fecundity bloodmeal volume and protein content

To estimate mosquito survival, a known number of mosquitoes were separated to a clean cardboard 3.8 L container. Dead mosquitoes were removed from the containers at the indicated time points and counted. The number of dead mosquitoes was subtracted from the total number of mosquitoes. To determine egg laying, single blood-fed mosquitoes were removed from the cages and placed in a single *Drosophila* tube containing water and sealed with a cotton ball. Mosquitoes were allowed to lay eggs for 1–2 days in a humidified 28°C room before being removed from the tubes. The eggs were counted under dissection microscope. The *Drosophila* tubes were incubated for an additional 1–2 days in the same conditions and the larvae were counted under dissection microscope. The percentage of larvae hatching was determined per the total number of eggs in the tube. For determination of bloodmeal, midguts were dissected immediately after blood feeding (2 cycles of 20 minutes per cage) and homogenized in 100 microliter PBS. A standard curve was made and hemoglobin was determined in spectrophotometer at a wavelength of 412 nM, as was done previously [56]. Similarly, bloodmeal protein contents were determined using the Lowry protein assay kit (Thermo # 23240) on midguts dissected immediately after blood feeding.

### Statistical analyses

Data were analyzed using GraphPad Prism 9.1.2 software. For normally distributed data, t-tests were used for paired and unpaired comparisons and one-way ANOVA for multiple treatments. For nonparametric data, the Mann–Whitney U test for pair-wise comparisons or Kruskal-Wallis test for multiple treatments were used. The specific test used for each experiment is indicated in the figure legends.

### Midgut bacterial loads

To assess the effect of adding antibiotics to the sucrose meal on the midgut bacterial loads, adult mosquitoes were fed with 15% sucrose with or without different concentrations of penicillin-streptomycin from the time of emergence. Midguts were dissected and homogenized in 100 microliters of sterile PBS. Serial dilutions of guts homogenates were then plated on LB-agar plates and colonies were counted to determine colony forming units (CFU).

### 16S ribosomal RNA gene amplicon sequencing

Genomic DNA was PCR amplified with primers 515F and 926R [57], targeting the V4 and V5 variable regions of the microbial small subunit ribosomal RNA gene using a two-stage “targeted amplicon sequencing (TAS)” protocol as described previously [21]. Primers were modified to include linker sequences at the 5’ ends (i.e., so-called “common sequences” or CS1 and CS2 on forward and reverse primers, respectively). First stage PCR amplifications were performed in 10 microliter reactions in 96-well plates, using the MyTaq HS 2X master mix (BioLine, Taunton, MA, USA). PCR conditions were 95°C for 5 minutes, followed by 28 cycles of 95°C for 30”, 50°C for 60” and 72°C for 90”. Subsequently, a second PCR amplification was performed in 10 microliter reactions in 96-well plates. A master mix for the entire plate was made using the MyTaq HS 2X master mix. Each well received a separate primer pair with a unique 10-base barcode, obtained from the Access Array Barcode Library for Illumina (Fluidigm, South San Francisco, CA; Item# 100–4876). These AccessArray primers contained the CS1 and CS2 linkers at the 3’ ends of the oligonucleotides. Cycling conditions were as follows: 95°C for 5 minutes, followed by 8 cycles of 95°C for 30”, 60°C for 30” and 72°C for 30”. A final, 7-minute elongation step was performed at 72°C. Samples were pooled in an equal volume using an EpMotion5075 liquid handling robot (Eppendorf, Hamburg, Germany). The pooled library was purified using an AMPure XP cleanup protocol (0.6X, vol/vol; Agencourt, Beckmann-Coulter) to remove fragments smaller than 300 bp. The pooled libraries, with a 20% phiX spike-in, were loaded onto an Illumina MiniSeq mid-output flow cell (2x150 paired-end reads). Based on the distribution of reads per barcode, the amplicons (before purification) were re-pooled to generate a more balanced distribution of reads. The re-pooled library was purified using AMPure XP cleanup, as described above. The re-pooled libraries, with a 15% phiX spike-in, were loaded onto a MiSeq v3 flow cell, and sequenced using an Illumina MiSeq sequencer. Fluidigm sequencing primers, targeting the CS1 and CS2 linker regions, were used to initiate sequencing. De-multiplexing of reads was performed on instrument. Library preparation, pooling, and sequencing were performed at the Genome Research Core, Research Resources Center (RRC), University of Illinois at Chicago (UIC).

Basic processing: Forward and reverse reads were merged using PEAR [58]. Merged reads were trimmed to remove ambiguous nucleotides, primer sequences, and trimmed based on quality threshold of p = 0.01. Reads that lacked either primer sequence and any sequences less than 300 bp were discarded. Chimeric sequences were identified and removed using the USEARCH algorithm with a comparison to Silva v132 reference sequence database [59].

The standard QIIME pipeline was modified to generate taxonomic summaries using sub-OTU resolution of the sequence dataset [60,61]. Briefly, the resulting sequence files were then merged with sample information. All sequences were then dereplicated to produce a list of unique sequences. All sequences that had an abundance of at least 10 counts were designated seed sequences. USEARCH was then used to find the nearest seed sequence for any non-seed sequence with a minimum identity threshold of 97%. For any non-seed sequence that matched a seed sequence, its counts were merged with the seed sequence counts [59]. For any non-seed sequence that did not match a seed sequence it would remain an independent sequence.

Taxonomic annotations for seed and unmatched non-seed sequences were assigned using the USEARCH and Silva v132 reference with a minimum similarity threshold of 90% [59,62]. In order to improve depth of annotation, the standard QIIME assignment algorithm was modified to only consider hits at each taxonomic level that had an assigned name. For example, a reference annotated as “k__Bacteria; p__Firmicutes; c__Clostridia; o__Clostridiales; f__Ruminococcaceae; g__; s__” would be considered in the assignment of the taxonomic kingdom through family, but would not be used for the assignment of the genus or species. Furthermore, any hits in the reference database must have a minimum identity of 97% or 99% to be considered for genus or species level assignment, respectively. Taxonomic annotations and sequence abundance data were then merged into a single sequence table.

Visualizations of the data were performed in the software package Primer7 (PRIMER 7, PRIMER-E Ltd. Lutton, UK). For multi-dimensional scaling analyses, data were log(x+1) transformed, and Bray-Curtis dissimilarity was calculated for all pairwise comparisons. Analysis of similarity (ANOSIM) was performed within Primer7 to determine if microbial communities were significantly different between groups; 999 permutations were used. Alpha diversity indices were also calculated in Primer7 using a rarefied dataset of 12,000 sequences/sample. Heatmaps were generated using the Matrix Display function in the Primer7 software package. Overall similarity of samples within each group were assessed a Mann-Whitney non-parametric test of Bray-Curtis dissimilarity values. Similarly, alpha diversity values (richness, evenness, Shannon index) were compared between groups using Mann-Whitney tests. Mann-Whitney tests were performed in the software package OriginPro (OriginLab Corporation). DESeq2 analysis, conducted in the software package DE Analysis App [63], was used to identify taxa significantly differently abundant between groups; p-values were adjusted with a false discovery rate correction. See dataset S1 for operational taxonomic units (OUT)

Raw sequence data (FASTQ files) were deposited in the National Center for Biotechnology Information (NCBI) Sequence Read Archive (SRA), under the BioProject identifier PRJNA767345.

## Supporting information

S1 Fig Generation of *Δaslrim1* mosquito line. (A) PCR strategy for detection of indels in mosquitoes during the CRISPR procedure. Primers 1 and 2 flank the expected deletion site (marked in red) and are used to amplify both the WT and the deletion alleles. The 3′ end of primer 3 (13 bp, marked in red) is anchored in the deletion and thus the primer should anneal only to the WT allele. One base pair of the 3′ end of primer 4 is anchored upstream to the deletion while the other 17 bp are anchored downstream for the deletion and thus PCR with this primer should only amplify the deletion allele. (B) Alignment showing Homozygous deletion of 13 nucleotides in *LRIM1* gene in 10 randomly collected *Δaslrim1* mosquitoes from G_12_. The alignment was done on sequences generated from PCR that was done with primers 1 and 2. (C) Diagnostic PCR amplifying the WT allele (upper panel, primers 1 and 3) and the deletion allele (lower panel, primers 1 and 4) in 2 WT and 10 randomly collected G_12_ *Δaslrim1* mosquitoes.
(TIFF)

S2 Fig Conformation of *LRIM1* knockout at the RNA and protein levels. (A) Upper panel -Scheme of the real-time PCR strategy. The nucleotide position indicated refers to the position of the primers on the *LRIM1* gene (ASTE000814). PCR I was done with Primers 5 and 6 (S6 Table) and targets a region downstream to the deletion and therefore should amplify both the WT and the Deletion alleles. PCR II is done with primers 7 and 8 and is aimed to amplify only the WT allele as the 3′ end of primer 5 is anchored in the deletion. Lower panel- Real-time PCR done with the above primer sets. Results are the mean mRNA abundance ± SD of 4 different replicate RNA samples from larvae, pupae and female and male adults. Each sample is a pool of 10 individuals from each of the life stages. The results are indicated as the *LRIM1* or *TEP1* mRNA abundance relative to the housekeeping, *S7* ribosomal protein gene (Primers 9 and 10, S6 Table). (B) Western blot analysis: Hemolymph from 7 female mosquitoes were loaded in each well in the gel and transferred to PVDF membranes. Membranes were reacted with anti 1:250 LRIM1 antiserum generated in this work. The expected molecular weight of LRIM1 protein is 58.3 kDa. For loading control, membrane was reacted with 1:500 AsTEP1 antiserum (ABBIOTECH # 250881).
(TIFF)

S3 Fig The effect of *LRIM1* deletion on bacterial loads, survival, bloodmeal volume and protein contents. (A) Real-time PCR quantification of the bacterial population densities in the mosquitoes. The reaction used 16S rDNA primers, 515F and 806R (Primers 13 and 14, S6 Table), targeting the bacterial V4 region of the SSU rDNA [52]. The PCR was done on individual non-fed females from WT and *Δaslrim1*. The results represent the mean ± SD (n = 10) of the bacterial load in *Δaslrim1* relative to WT. Mosquito ribosomal protein gene S*7* was used as a housekeeping gene. (B) Survival rate of WT and *Δaslrim1* mosquitoes grown on 15% sugar with or without PS. Females were provided with bloodmeal and 4 days following blood feeding the number of dead mosquitoes in each cage was determined. The % survival is determined by the number of live mosquitoes relative to the number of mosquitoes placed in each cage. The results represent the mean ± SD of the % surviving mosquitoes in each cage (n = 3). The data was analyzed by one-way ANOVA. (C) Bloodmeal volumes in WT and *Δaslrim1* mosquitoes immediately after blood feeding. The results represent the mean of bloodmeal volumes ± SD; n = 21. (D) Bloodmeal protein contents in midguts of WT and *Δaslrim1* mosquitoes, immediately after bloodmeal. The protein contents were determined using the Lowry protein assay. The results represent the mean of midgut protein contents in micrograms ± SD; n = 21. (C and D) The results were analyzed using unpaired t-test. In the entire figure NS P>0.05, *P≤0.05, P** P≤0.01, ***P≤0.001, ****P≤0.0001.
(TIFF)

S4 Fig Alpha diversity and analysis of similarity in WT and *Δaslrim1* mosquitoes. (A-B) Comparison of alpha diversity. (A) Shannon index (log base e) of microbial communities in WT and *Δaslrim1* mosquitoes. (B) Microbial community evenness. The differences in both measures were not statistically significant (MWU, P = 0.4034 and MWU, P = 1 in A and B, respectively). Alpha diversity indices were calculated on rarefied datasets (12,000 sequences/sample). (C) Metric Multidimensional Scaling (mMDS) plot of Mosquito-associated microbial communities. Analysis was performed at the taxonomic level of genus. Data was log(x+1) transformed, and Bray-Curtis similarity was calculated for all pairwise comparisons. (D) Bray-Curtis similarity values were represented in two dimensions, and 2D stress was 0.08. Analysis of similarity (ANOSIM) indicated that microbial communities between the two mosquito lines were significantly different (R = 0.524, p = 0.008).
(TIFF)

S5 Fig The effect of Pen Strep addition to the sucrose meal on midgut bacterial loads, survival and fecundity. (A) Survival of WT mosquitoes maintained on 0%, 0.5% and 1% (v/v) of Penicillin-Streptomycin (PS) solution (500 U/mL), diluted in 15% sucrose. The survival Percentage is the number of live mosquitoes in each time point relative to the number of mosquitoes put in the cage on day zero (30). (B) Mean ±SD of the number of eggs per females grown on different PS concentrations. The mosquitoes were provided with bloodmeal 3 days after transferring them to cages and individual mosquitoes were put in *Drosophila* tubes for oviposition 4 days after bloodmeal. Eggs were counted in each tube. (C) The number of larvae in each *Drosophila* tube was determined 1-2 days post oviposition. The results show the % of larvae out of the total number of eggs laid in that particular tube. (B, C) n = 4, 5 and 5 for 0%, 0.5% and 1% PS, respectively. (D) Colony forming units (CFU) in individual guts of WT mosquitoes in different PS concentrations. Each gut was diluted 10 and 100 times in sterile PBS and the CFU for each gut is the mean between the two dilutions. The results show the mean CFU for different guts (n = 4, 3 and 3 for 0%, 0.5% and 1%, respectively). (E) Colony forming units (CFU) in individual gut of WT and *Δaslrim1* mosquitoes grown with and without 1%PS (P≤0.05). Each gut was diluted 10, 100 and 1000 times in sterile PBS and the CFU for each gut is the mean between the three dilutions. The results show the mean ±SD CFU for different guts (n = 4).
(TIFF)

S1 Table Short guide RNAs. (PDF)

S2 Table CRISPR efficiency. (PDF)

S3 Table Fecundity in wild-type and *Δaslrim1 Anopheles stephensi* grown on 15% sucrose with or without pen strep. (PDF)

S4 Table Fecundity in WT and *Δaslrim1* grown W/O pen strep. (PDF)

S5 Table Oviposition and egg production in WT females inseminated by WT or *Δaslrim1* males. (PDF)

S6 Table Primers used in this work. (PDF)

S7 Table Accession numbers in NCBI Sequence Read Archive (SRA) depository of FASTQ files for 16S amplicon sequencing analysis done on 5 WT and 5 *Δaslrim1* (KO) mosquitoes. Bioproject ID: PRJNA767345
(PDF)

S1 Dataset Operational taxonomic unit (OTU). (XLSX)
