# Supplementary figures and images for: Knockout of Anopheles stephensi immune gene LRIM1 by CRISPR-Cas9 reveals its unexpected role in reproduction and vector competence
Source: PLoS Pathog. 2021 Nov 16;17(11):e1009770. doi: 10.1371/journal.ppat.1009770 (PMC8631644; doi:10.1371/journal.ppat.1009770)

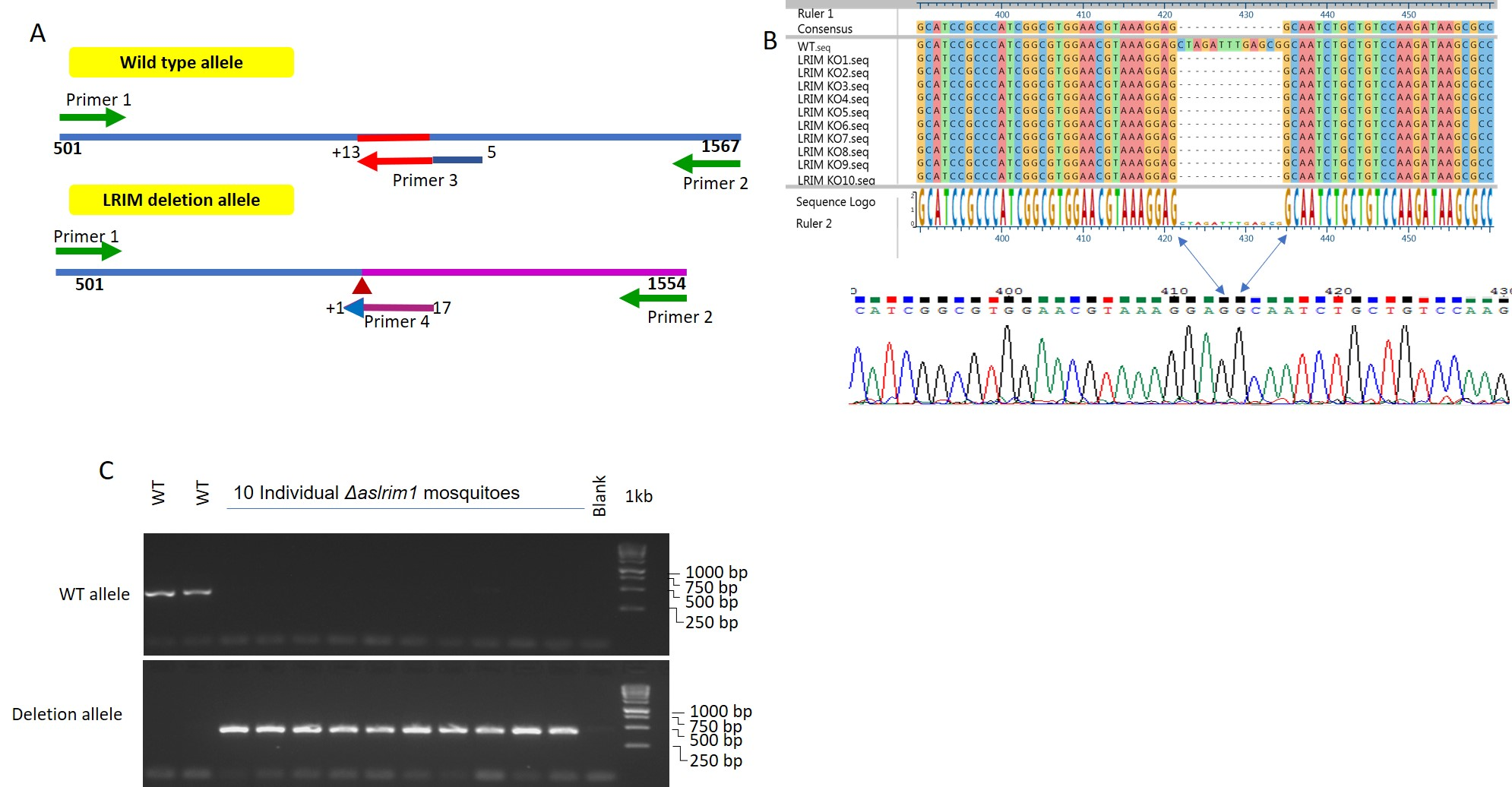

Supplement: S1 Fig — (A) PCR strategy for detection of indels in mosquitoes during the CRISPR procedure. Primers 1 and 2 flank the expected deletion site (marked in red) and are used to amplify both the WT and the deletion alleles. The 3′ end of primer 3 (13 bp, marked in red) is anchored in the deletion and thus the primer should anneal only to the WT allele. One base pair of the 3′ end of primer 4 is anchored upstream to the deletion while the other 17 bp are anchored downstream for the deletion and thus PCR with this primer should only amplify the deletion allele. (B) Alignment showing Homozygous deletion of 13 nucleotides in LRIM1 gene in 10 randomly collected Δaslrim1 mosquitoes from G12. The alignment was done on sequences generated from PCR that was done with primers 1 and 2. (C) Diagnostic PCR amplifying the WT allele (upper panel, primers 1 and 3) and the deletion allele (lower panel, primers 1 and 4) in 2 WT and 10 randomly collected G12 Δaslrim1 mosquitoes. (TIFF) [file ppat.1009770.s001.tiff]

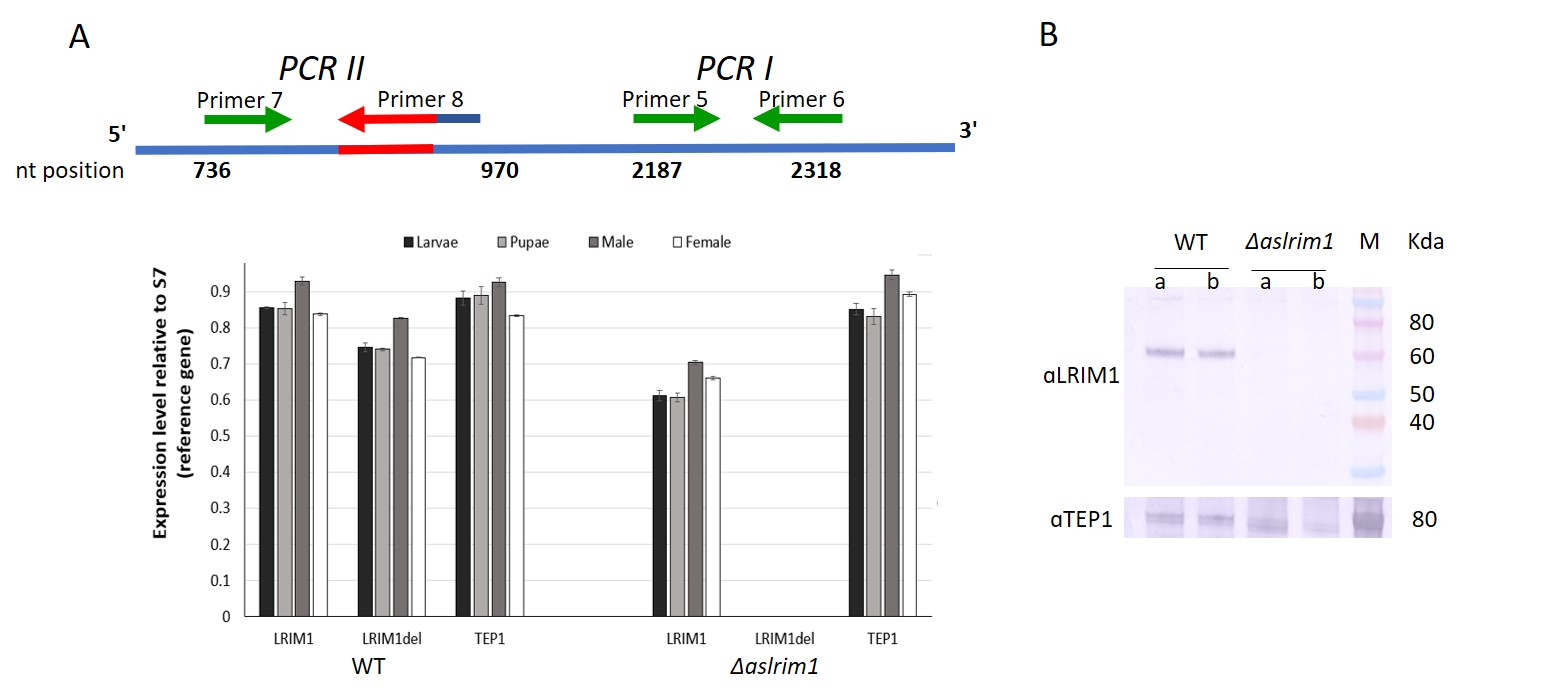

Supplement: S2 Fig — (A) Upper panel -Scheme of the real-time PCR strategy. The nucleotide position indicated refers to the position of the primers on the LRIM1 gene (ASTE000814). PCR I was done with Primers 5 and 6 (S6 Table) and targets a region downstream to the deletion and therefore should amplify both the WT and the Deletion alleles. PCR II is done with primers 7 and 8 and is aimed to amplify only the WT allele as the 3′ end of primer 5 is anchored in the deletion. Lower panel- Real-time PCR done with the above primer sets. Results are the mean mRNA abundance ± SD of 4 different replicate RNA samples from larvae, pupae and female and male adults. Each sample is a pool of 10 individuals from each of the life stages. The results are indicated as the LRIM1 or TEP1 mRNA abundance relative to the housekeeping, S7 ribosomal protein gene (Primers 9 and 10, S6 Table). (B) Western blot analysis: Hemolymph from 7 female mosquitoes were loaded in each well in the gel and transferred to PVDF membranes. Membranes were reacted with anti 1:250 LRIM1 antiserum generated in this work. The expected molecular weight of LRIM1 protein is 58.3 kDa. For loading control, membrane was reacted with 1:500 AsTEP1 antiserum (ABBIOTECH # 250881). (TIFF) [file ppat.1009770.s002.tiff]

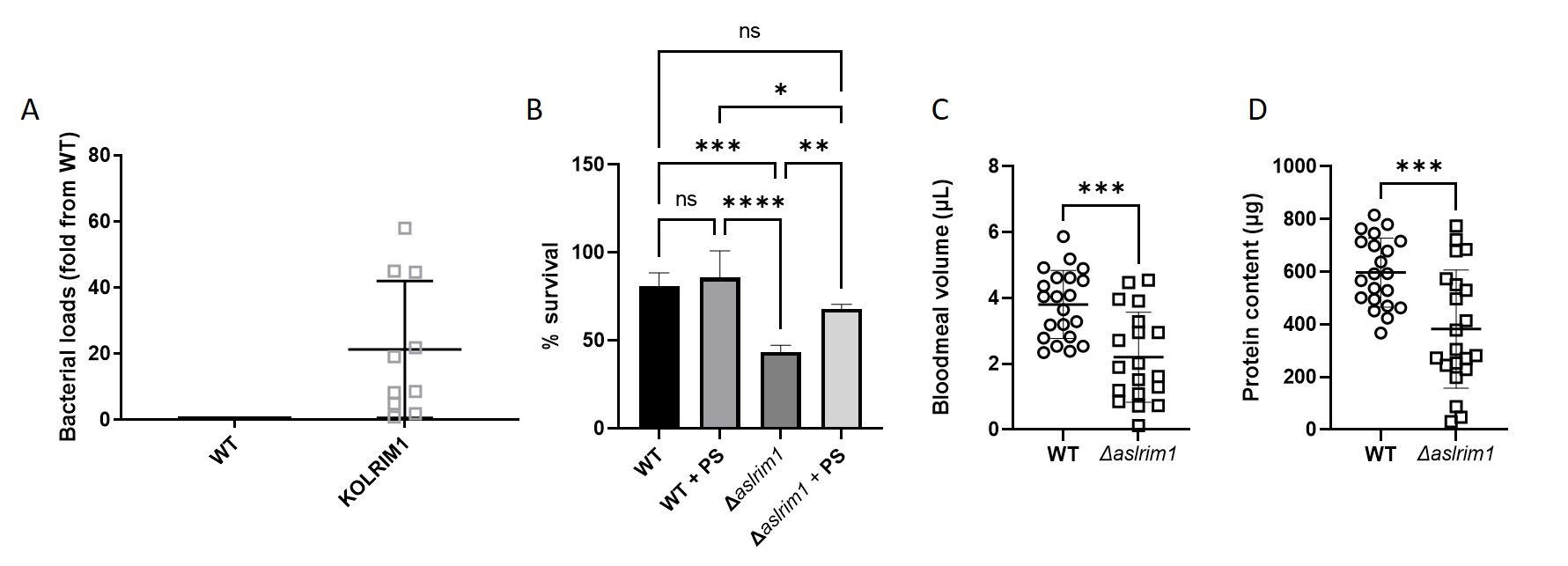

Supplement: S3 Fig — (A) Real-time PCR quantification of the bacterial population densities in the mosquitoes. The reaction used 16S rDNA primers, 515F and 806R (Primers 13 and 14, S6 Table), targeting the bacterial V4 region of the SSU rDNA [52]. The PCR was done on individual non-fed females from WT and Δaslrim1. The results represent the mean ± SD (n = 10) of the bacterial load in Δaslrim1 relative to WT. Mosquito ribosomal protein gene S7 was used as a housekeeping gene. (B) Survival rate of WT and Δaslrim1 mosquitoes grown on 15% sugar with or without PS. Females were provided with bloodmeal and 4 days following blood feeding the number of dead mosquitoes in each cage was determined. The % survival is determined by the number of live mosquitoes relative to the number of mosquitoes placed in each cage. The results represent the mean ± SD of the % surviving mosquitoes in each cage (n = 3). The data was analyzed by one-way ANOVA. (C) Bloodmeal volumes in WT and Δaslrim1 mosquitoes immediately after blood feeding. The results represent the mean of bloodmeal volumes ± SD; n = 21. (D) Bloodmeal protein contents in midguts of WT and Δaslrim1 mosquitoes, immediately after bloodmeal. The protein contents were determined using the Lowry protein assay. The results represent the mean of midgut protein contents in micrograms ± SD; n = 21. (C and D) The results were analyzed using unpaired t-test. In the entire figure NS P>0.05, *P≤0.05, P** P≤0.01, ***P≤0.001, ****P≤0.0001. (TIFF) [file ppat.1009770.s003.tiff]

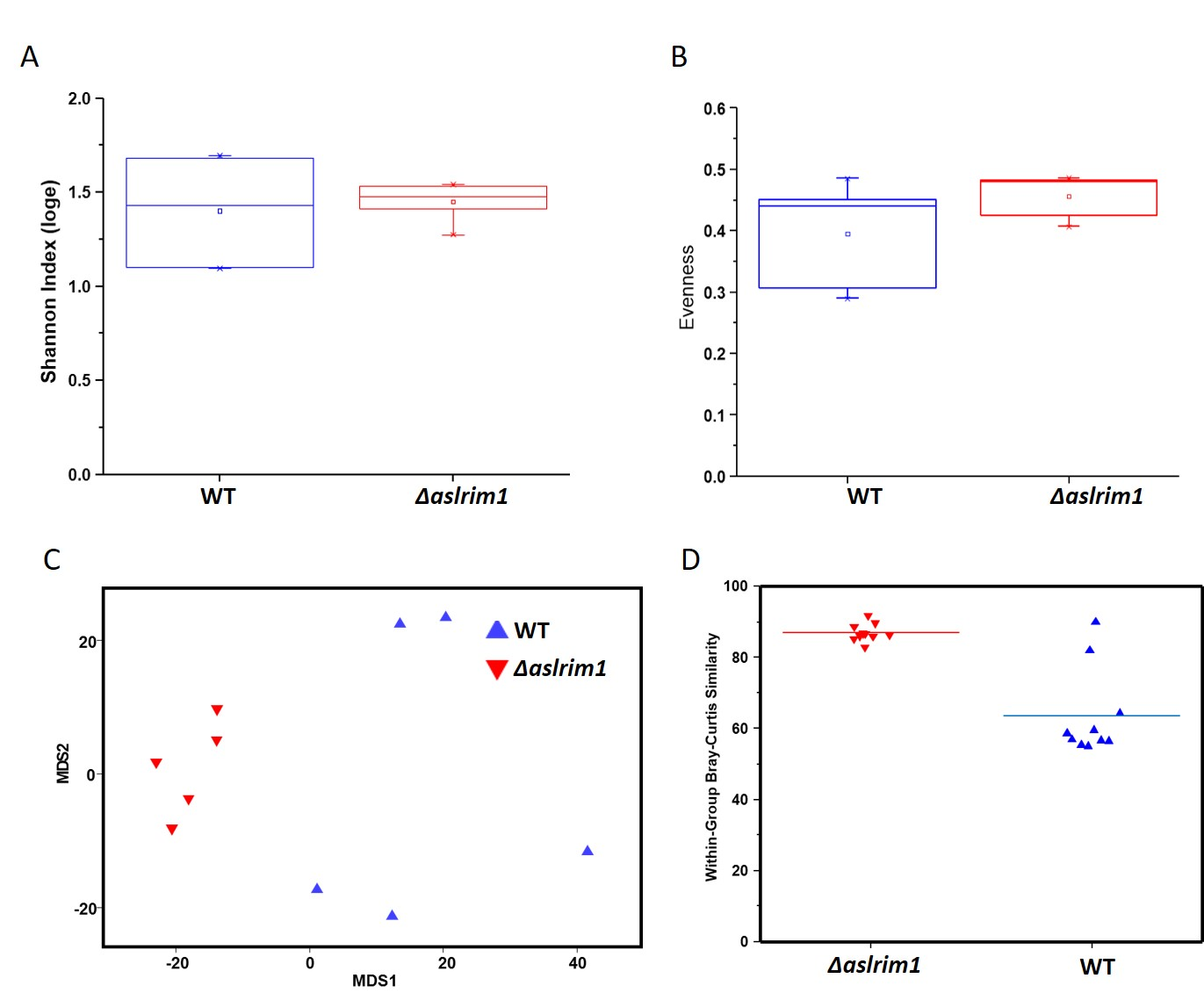

Supplement: S4 Fig — (A-B) Comparison of alpha diversity. (A) Shannon index (log base e) of microbial communities in WT and Δaslrim1 mosquitoes. (B) Microbial community evenness. The differences in both measures were not statistically significant (MWU, P = 0.4034 and MWU, P = 1 in A and B, respectively). Alpha diversity indices were calculated on rarefied datasets (12,000 sequences/sample). (C) Metric Multidimensional Scaling (mMDS) plot of Mosquito-associated microbial communities. Analysis was performed at the taxonomic level of genus. Data was log(x+1) transformed, and Bray-Curtis similarity was calculated for all pairwise comparisons. (D) Bray-Curtis similarity values were represented in two dimensions, and 2D stress was 0.08. Analysis of similarity (ANOSIM) indicated that microbial communities between the two mosquito lines were significantly different (R = 0.524, p = 0.008). (TIFF) [file ppat.1009770.s004.tiff]

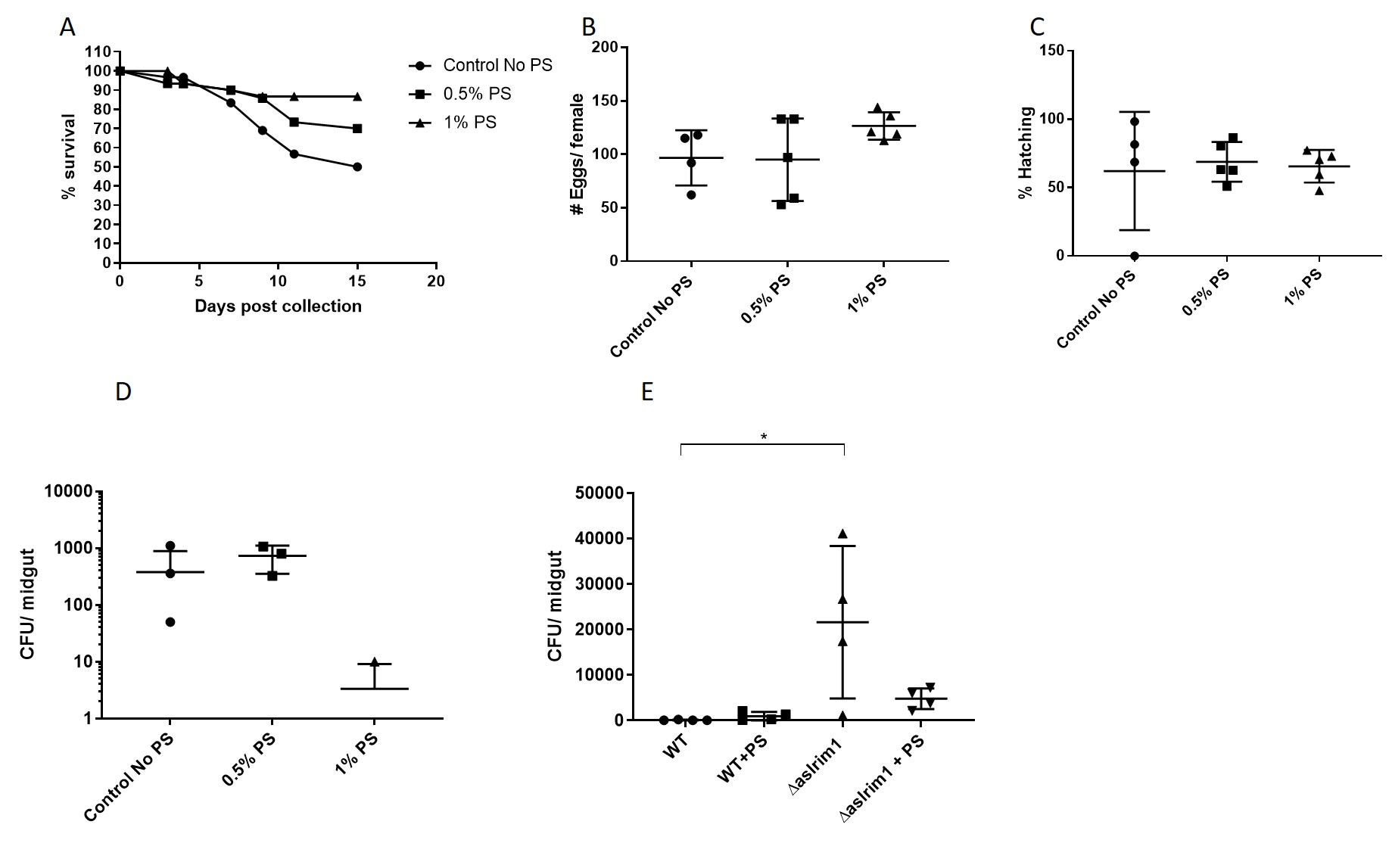

Supplement: S5 Fig — (A) Survival of WT mosquitoes maintained on 0%, 0.5% and 1% (v/v) of Penicillin-Streptomycin (PS) solution (500 U/mL), diluted in 15% sucrose. The survival Percentage is the number of live mosquitoes in each time point relative to the number of mosquitoes put in the cage on day zero (30). (B) Mean ±SD of the number of eggs per females grown on different PS concentrations. The mosquitoes were provided with bloodmeal 3 days after transferring them to cages and individual mosquitoes were put in Drosophila tubes for oviposition 4 days after bloodmeal. Eggs were counted in each tube. (C) The number of larvae in each Drosophila tube was determined 1-2 days post oviposition. The results show the % of larvae out of the total number of eggs laid in that particular tube. (B, C) n = 4, 5 and 5 for 0%, 0.5% and 1% PS, respectively. (D) Colony forming units (CFU) in individual guts of WT mosquitoes in different PS concentrations. Each gut was diluted 10 and 100 times in sterile PBS and the CFU for each gut is the mean between the two dilutions. The results show the mean CFU for different guts (n = 4, 3 and 3 for 0%, 0.5% and 1%, respectively). (E) Colony forming units (CFU) in individual gut of WT and Δaslrim1 mosquitoes grown with and without 1%PS (P≤0.05). Each gut was diluted 10, 100 and 1000 times in sterile PBS and the CFU for each gut is the mean between the three dilutions. The results show the mean ±SD CFU for different guts (n = 4). (TIFF) [file ppat.1009770.s005.tiff]
