# Supplementary material for: Knockout of Anopheles stephensi immune gene LRIM1 by CRISPR-Cas9 reveals its unexpected role in reproduction and vector competence
Source: PLoS Pathog. 2021 Nov 16;17(11):e1009770. doi: 10.1371/journal.ppat.1009770 (PMC8631644; doi:10.1371/journal.ppat.1009770)
Supplement: S1 Table — (PDF) [file ppat.1009770.s006.pdf]

Table S1. Short guide RNAs

Short guide	Sequence	Predicted cleavage efficiency	Protospacer adjacent motif (PAM) starts at gene position (bp)	Exon
1	GGAAGAAACACGTTGAACAAGA	0.53	712	1
2	GGTGAACAAGATGGTGTCGTTG	0.54	723	1
3	GGAAAGCATCCGCCCATCGGCG	0.58	923	2
4	GGCCCATCGGCGTGGAACGTAA	0.46	938	2
5	GGTAAAGGAGCTAGATTTGAG	0.55	954	2
