# Supplementary material for: Knockout of Anopheles stephensi immune gene LRIM1 by CRISPR-Cas9 reveals its unexpected role in reproduction and vector competence
Source: PLoS Pathog. 2021 Nov 16;17(11):e1009770. doi: 10.1371/journal.ppat.1009770 (PMC8631644; doi:10.1371/journal.ppat.1009770)
Supplement: S2 Table — (PDF) [file ppat.1009770.s007.pdf]

Table S2. CRISPR efficiency

**CAS9 concentrations (ng/µL)**	**# eggs injected**	**# eggs hatched**	**G_0**		**G_1**		**Indels**	
			♀	♂	♀	♂	♀	♂
300	549	19	4	6	4	16	0	1
