# Supplementary material for: Knockout of Anopheles stephensi immune gene LRIM1 by CRISPR-Cas9 reveals its unexpected role in reproduction and vector competence
Source: PLoS Pathog. 2021 Nov 16;17(11):e1009770. doi: 10.1371/journal.ppat.1009770 (PMC8631644; doi:10.1371/journal.ppat.1009770)
Supplement: S3 Table — (PDF) [file ppat.1009770.s008.pdf]

Table S3. Fecundity in wild-type and *Δaslrim1 Anopheles stephensi* grown on 15% sucrose with or without pen strep

**Group**	**Number of females**	**Proportion of females ovipositing (%)**	**Geometric mean number of eggs laid per female (95% Confidence interval)**	**Geometric mean % Hatching (95% Confidence interval)**
WT	34	95.2	111.1 (104.2-126)	52.9 (37.5-74.5)
WT + PS	35	84.9	97.6 (92.3-114.1)	72.4 (66.7-78.6)
Δaslrim1	34	61.4	55.7 (53.1-68.7)	22.8 (13.5-38.7)
Δaslrim1 + PS	35	55.4	60.9 (57.1-70.3)	25 (17.5-36)
