# Supplementary material for: Knockout of Anopheles stephensi immune gene LRIM1 by CRISPR-Cas9 reveals its unexpected role in reproduction and vector competence
Source: PLoS Pathog. 2021 Nov 16;17(11):e1009770. doi: 10.1371/journal.ppat.1009770 (PMC8631644; doi:10.1371/journal.ppat.1009770)
Supplement: S4 Table — (PDF) [file ppat.1009770.s009.pdf]

Table S4. Fecundity in WT and *Δaslrim1* grown W/O pen strep

Line/Treatment	Cage	# Females placed in the cage	# Females survived at the day of oviposition	% Survival[1]	# Eggs/ cage	# Eggs/ female
**WT**	A	**30**	22	73.3	276	12.5
	B	**30**	27	90	733	27.1
	C	**30**	27	90	92	3.4
	**Average**			**84.4**	**367**	**14.4**
	**SD[2]**			**9.6**	**330**	**11.9**
**WT + PS**	A	**30**	23	76.6	1021	44.3
	B	**30**	18	60	549	30.5
	C	**30**	22	73.3	302	13.7
	**Average**			**70**	**624**	**29.5**
	**SD**			**8.8**	**365.3**	**15.4**
Δaslrim1	A	**30**	7	23.3	0	0
	B	**30**	6	20	0	0
	C	**30**	5	16.6	0	0
	**Average**			**20**	**0**	**0**
	**SD**			**3.3**	**0**	**0**
***Δaslrim1* +PS**	A	**30**	18	60	0	0
	B	**30**	18	60	0	0
	C	**30**	13	43.3	0	0
	**Average**			**54.4**	**0**	**0**
	**SD**			**9.6**	**0**	**0**

[1] – The results in this column are presented in figure S3B; SD= Standard deviation
