# Supplementary material for: Knockout of Anopheles stephensi immune gene LRIM1 by CRISPR-Cas9 reveals its unexpected role in reproduction and vector competence
Source: PLoS Pathog. 2021 Nov 16;17(11):e1009770. doi: 10.1371/journal.ppat.1009770 (PMC8631644; doi:10.1371/journal.ppat.1009770)
Supplement: S5 Table — (PDF) [file ppat.1009770.s010.pdf]

Table S5. Oviposition and egg production in WT females inseminated by WT or *Δaslrim1* males

Cross	#Live females[1]	# Females laying eggs	% Females laying eggs	% Females with eggs in the ovaries[2]	# Eggs/female[3]
♂WT X ♀WT	28	9	32	17	106±21
♂*Δaslrim1* X ♀WT	29	2	7	42	118±11

[1] – Live females were determined at the day of egg laying; [2]- Percentage is calculated from the number of females that did not lay eggs; [3]- The average ± SD of eggs/female was calculated from the females that laid eggs.
