# Supplementary material for: Knockout of Anopheles stephensi immune gene LRIM1 by CRISPR-Cas9 reveals its unexpected role in reproduction and vector competence
Source: PLoS Pathog. 2021 Nov 16;17(11):e1009770. doi: 10.1371/journal.ppat.1009770 (PMC8631644; doi:10.1371/journal.ppat.1009770)
Supplement: S6 Table — (PDF) [file ppat.1009770.s011.pdf]

Table S6. Primers used in this work

Primer	Primer target	Primer sequence
1	5’ flank of the deletion	CCACAACTCTCTGCGGTGTA
2	3’ flank of the deletion	GTGACAGCGGGTGAAAACTT
3	Reverse primer, anchored in the deletion, amplifying WT allele	CAGCAGATTGCCTCCTTTA
4	Reverse primer, flanking both sides of the deletion, amplifying deletion allele	AGATTGCCGCTCAAATCTA
5	Forward primer, downstream to the deletion, for amplification of the LRIM1 transcript in WT and in the deletion alleles	GAGGAAAATGCTCGGATGAA
6	Reverse primer, downstream to the deletion, for amplification of the LRIM1 transcript in WT and in the deletion alleles	CGACGGCTGAACCTTACTGA
7	Forward primer upstream to the deletion for amplification of the WT allele	TTTGCTAGCGTTCGTTTGTG
8	Reverse primer, anchored in the deletion, amplifying the WT allele only	AGATTGCCGCTCAAATCTA
9	S7, mosquito rDNA forward	TTCGTTGTGAACCCAAATAAAAATC

10	S7, mosquito rDNA Reverse	TGCGGCTTCAGATCCGAGTTC
11	Forward primer for qPCR, amplifying TEP1 transcript	TTGCTGTCGTTCGTGATA
12	Reverse primer for qPCR, amplifying TEP1 transcript	AGCGTGATGGTGTAGTCG
13	16S-rDNA, 515F	GTGCCAGCMGCCGCGGTAA
14	16S-rDNA, 806R	GGACTACHVGGGTWTCTAAT
