# Supplementary material for: Knockout of Anopheles stephensi immune gene LRIM1 by CRISPR-Cas9 reveals its unexpected role in reproduction and vector competence
Source: PLoS Pathog. 2021 Nov 16;17(11):e1009770. doi: 10.1371/journal.ppat.1009770 (PMC8631644; doi:10.1371/journal.ppat.1009770)
Supplement: S7 Table — Bioproject ID: PRJNA767345 (PDF) [file ppat.1009770.s012.pdf]

Table S7. Accession numbers in NCBI Sequence Read Archive (SRA) depository of FASTQ files for 16S amplicon sequencing analysis done on 5 WT and 5 *Δaslrim1* (KO) mosquitoes. Bioproject ID: PRJNA767345

Accession	Sample Name
SAMN21898833	WT1
SAMN21898834	WT2
SAMN21898835	WT3
SAMN21898836	WT4
SAMN21898837	WT5
SAMN21898838	KO1
SAMN21898839	KO2
SAMN21898840	KO3
SAMN21898841	KO4
SAMN21898842	KO5
